# *Xenopsylla buxtoni* fleas as a dominant species harboring multiple infections of *Wolbachia* lineages in the ancient plague epicenters of Iran

**DOI:** 10.1371/journal.pntd.0013890

**Published:** 2026-02-13

**Authors:** Shahin Seidi, Ehsan Mostafavi, Abbasali Raz, Fateh Karimian, Fariba Khanzadeh, Zeynab Hayati, Naseh Maleki-Ravasan

**Affiliations:** 1 Department of Epidemiology and Biostatics, Research Centre for Emerging and Reemerging Infectious Diseases, WHO Collaborating Center for Vector-Borne Diseases, Pasteur Institute of Iran, Tehran, Iran; 2 National Reference Laboratory for Plague, Tularemia and Q fever, Research Centre for Emerging and Reemerging Infectious Diseases, Pasteur Institute of Iran, Akanlu, Iran; 3 Malaria and Vector Research Group, Biotechnology Research Center, Pasteur Institute of Iran, Tehran, Iran; 4 Department of Parasitology, Pasteur Institute of Iran, Tehran, Iran; 5 Department of Zoology, Comenius University, Bratislava, Slovakia; Lanzhou University, CHINA

## Abstract

Fleas are permissive and euryxenous ectoparasites capable of transmitting numerous ancient and new pathogens among warm-blooded animals, including humans. Precise identification of flea species involved in disease transmission and understanding the highly specialized morphological characteristics associated with their ectoparasitic lifestyle is essential. Likewise, identifying endosymbionts such as *Wolbachia*—which have long-lasting and intimate relationships with their hosts—will enhance our knowledge of the epidemiology of flea-borne diseases and their control. Flea sampling was conducted in the western half of Iran, where the highest plague outbreaks have been reported over the past two centuries. A total of 1,439 fleas, comprising 623 males and 816 females, were detached from 223 hosts and were identified as *Xenopsylla buxtoni*, *X. nuttalli*, *X. astia*, *Pulex irritans*, *Nosopsyllus iranus iranus*, and *Ctenophthalmus rettigi smiti*. Also, 116 and 73 nucleotide sequences were analyzed to assess the genetic diversity and phylogenetic position of the fleas, and to determine their infection rate and *Wolbachia* supergroup. Molecular analysis of the *COII* and *ITS2* genes confirmed the morphological distinctiveness of the six species. *Xenopsylla buxtoni*, the most abundant taxon, displayed *Wolbachia* infection rates of 62%-75% (x̄ = 69%). The *Wolbachia* sequences identified from the fleas were assigned to supergroups A, F, and B. The taxonomic position of *X. buxtoni* and its closely related species, *X. nuttalli*, in the conformis group was questioned due to significant genetic divergence. The impact of *Wolbachia* on flea ecology and its potential impact in controlling flea populations and flea-borne pathogens was highlighted.

## Author summary

Fleas are important vectors of zoonotic diseases, including plague caused by *Yersinia pestis*. We investigated flea diversity and the presence of *Wolbachia* endosymbionts in western Iran—a region with a long history of plague outbreaks. Among 1,439 fleas collected from 223 hosts, six species were identified, with *Xenopsylla buxtoni* being the most common and showing high *Wolbachia* infection rates (62%–75%). Phylogenetic analysis revealed the presence of multiple *Wolbachia* supergroups (A, B, F), challenging existing assumptions about flea–symbiont co-evolution. We also observed significant genetic divergence between *X. buxtoni* and the closely related *X. nuttalli*, suggesting possible cryptic species that could complicate plague surveillance. Our findings highlight the ecological role of *Wolbachia* in fleas and its potential for use in targeted biocontrol strategies to reduce disease transmission. This study underscores the need to integrate symbiont-focused research into public health initiatives, particularly in plague-endemic areas.

## Introduction

Fleas (Siphonaptera) are blood-sucking, laterally compressed ectoparasites that can infest a wide range of hosts, including humans, due to their jumping ability. Fleas as solenophagous (vessel feeder) insects, can directly damage their host’s blood vessels and, more importantly, spread various pathogens via the oral route through regurgitation of blood meals or the fecal route by fecal pellets [1,2]. Because of their morphological adaptations, ecological plasticity, and global distribution [3,4], fleas play significant and effective roles as vectors for several human diseases, including plague, murine typhus, flea-borne spotted fever, and cat scratch disease, which are caused by *Y. pestis*, *Rickettsia typhi*, *R. felis*, and *Bartonella henselae*, respectively. They also serve as intermediate hosts for tapeworms of *Dipylidium caninum*, *Hymenolepis nana*, and *H. diminuta* [2]. Because competent vectors and reservoir hosts persist in endemic areas, understanding flea biodiversity and the factors affecting their vectorial capacity is critical for public health surveillance and disease control.

Fleas comprise over 2,600 documented species globally [5], and their precise identification is critical due to their role as vectors of zoonotic pathogens. Traditional taxonomy has relied on morphological traits such as genital structures, setae, spines, ctenidia, and other diagnostic features [6–11], but this approach is often challenging—particularly in females—due to subtle variations in key structures and a high degree of morphological specialization linked to their ectoparasitic lifestyle [12,13]. To overcome these limitations, molecular markers have increasingly been integrated into flea systematics. Commonly used insect phylogenetic markers—including cytochrome c oxidase subunits I and II (*COI* and *COII*), *12S* and *16S* ribosomal DNA (*rDNA*), *18S* and *28S rDNA*, internal transcribed spacer (*ITS*), and *EF-1a* [13–24], offer valuable tools for refining species boundaries. The ITS2 region exhibits considerable interspecific variability due to its high evolutionary rate [25], whereas *COII* shows strong concordance with morphological data in resolving phylogenetic relationships [23].

Endosymbiont are microbes that live inside hosts and maintain in long-term, intimate relationships with these microorganisms—may influence various aspects of host biology and behavior [26,27]. *Wolbachia*, a prominent intracellular bacterium, significantly impacts reproductive traits such as cytoplasmic incompatibility, male killing, feminization, and parthenogenesis. It al so contributes to fitness and lifespan, and plays roles in protect against biotic and abiotic stresses, immune hemostasis, host selection and mating behaviors, pathogen transmission capabilities, and host ecology and evolution [28–32]. *Wolbachia* is primarily an endosymbiotic bacterium prevalent in insects; however, it is also found in other arthropods and nematodes. Despite their genetic diversity, all known strains are currently classified within the species *Wolbachia pipientis* and are grouped into 16–21 major clades—referred to as supergroups—based on genetic characteristics identified in various studies [33]. Supergroups A and B are common in arthropods, while supergroups C, D, and J are specific to nematodes. Supergroup F is mainly found in nematodes but can also be observed in some terrestrial arthropods [34].

With the established background, it is evident that fleas and their associated *Wolbachia* bacteria serve as a valuable model for investigating the interplay between these microorganisms and their hosts. *Wolbachia* is highly prevalent in flea populations [35], indicating its significant potential to influence flea reproductive success and, consequently, the transmission of flea-borne diseases. The detection of these three supergroups (A, B, and F) in flea species provides insight into the complex evolutionary history and ecological interactions of *Wolbachia* within their flea hosts and potentially their rodent reservoirs. While numerous factors are known to influence flea reproductive ecology (e.g., body size, age, and host density), the role of *Wolbachia* has been largely overlooked, particularly in feral flea populations. Understanding how *Wolbachia* influences flea biology and pathogen transmission could inform future vector surveillance and pave the way for *Wolbachia*-based biocontrol strategies, though targeted research is needed to validate these applications.

Fleas, with 119 species and subspecies in Iran [36–39], pose a significant public health concern serving as vectors for various zoonotic diseases. The Pulicidae family, particularly the genus *Xenopsylla*, dominates the Iranian flea fauna and, along with other fleas, has been responsible for plague outbreaks in in past centuries [40]. During these outbreaks, the primary reservoirs and vectors were not always clearly identified or described. In the present study, hosts considered include rodents such as *Meriones persicus* and *Microtus arvalis*, which are known plague reservoirs, as well as foxes, which are also recognized as potential hosts and reservoirs in endemic areas. Given the continued presence of competent vectors and reservoir hosts in these regions, the risk of plague and other zoonotic diseases persists. A thorough understanding of flea biodiversity, the challenges in their morphological identification, and the potential impact of symbionts such as *Wolbachia* is therefore critical for enhancing surveillance and developing effective biocontrol strategies.

This study aimed to investigate the genetic diversity and phylogenetic relationships of key flea species in Iran’s ancient plague foci and to characterize their associations with *Wolbachia* endosymbionts. We used the nuclear *ITS2* and mitochondrial *COII* markers to assess genetic structure within and among flea taxa and to evaluate its congruence with morphological characteristics and geographic origin. Additionally, we analyzed *Wolbachia*-specific genes (*wsp*, *groEL*, and *gatB*) to determine infection prevalence and assign bacterial strains to supergroups.

We hypothesized that (i) flea community composition and genetic structure differ between plague-endemic and non-endemic regions, (ii) *Wolbachia* prevalence and strain diversity vary among flea species and geographic locations, and (iii) patterns of *Wolbachia* infection are associated with the distribution of flea species. By integrating phylogenetic, genetic, and infection data across spatially distinct sites, this study aims to advance our understanding of flea bioecology, elucidate the potential role of *Wolbachia* in vector dynamics, and evaluate the feasibility of *Wolbachia*-based strategies for vector control.

## Materials and methods

### Ethics statement

The study procedure has been reviewed and approved by the Ethics Committee of the Pasteur Institute of Iran, Tehran (ethical code: IR.PII.REC.1400.047). All animal procedures were conducted in accordance with the European Community Directive 2010/63/EU on the protection of animals used for scientific purposes.

### Description of study areas and specimen collection

**Fig 1:**
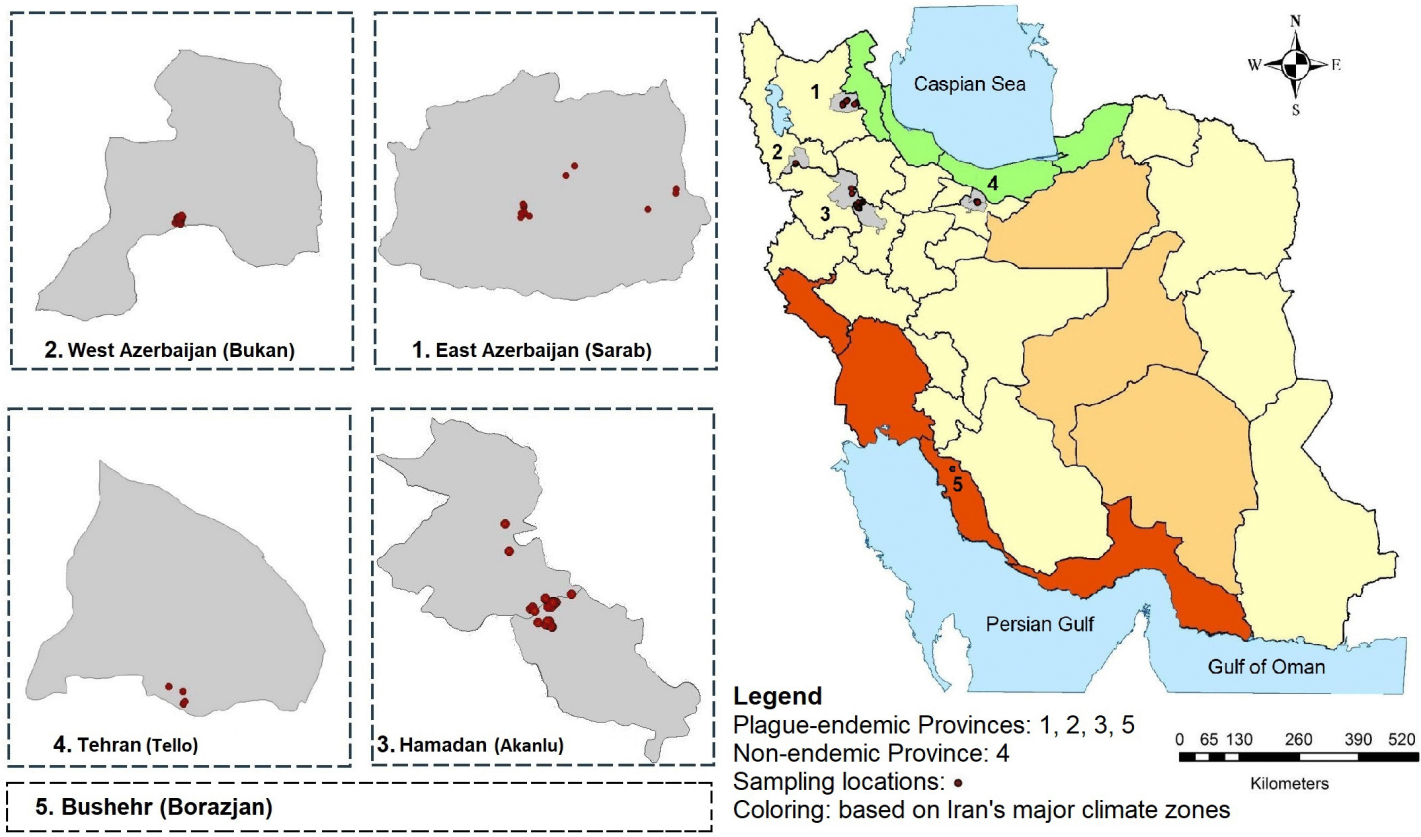
A map of flea sampling locations indicating areas of plague occurrence (East and West Azerbaijan, Hamadan, Bushehr Provinces) and non-occurrence (Tehran Province) in Iran (https://www.amar.org.ir).

Flea sampling was focused on the western half of Iran, where the highest outbreaks of plague have been reported over the past two centuries [41]. The most significant outbreak occurred in the Kurdistan region, where three forms of the plague—pneumonic, bubonic, and septicemic—were recorded. During the sylvatic outbreak of 1829–1833, *Meriones* spp. and their fleas were identified as the reservoir and vector hosts of the plague, respectively [42–44]. This study selected 28 locations across 10 villages to trap rodents and collect their fleas, covering both the historical focus and neighboring villages (Fig 1). Another plague outbreak occurred in 1966 in Seyed-abad, southwest of Bukan in the West Azerbaijan Province of the country. Although the vector flea and reservoir host of the disease were not identified during this outbreak, evidence suggests the presence of the bubonic form of the disease [45]. Fifteen locations from two villages were selected for flea investigation in this focus (Fig 1). The third major outbreak of the plague occurred in various villages west of Sarab in East Azerbaijan Province in 1976. No human cases were reported during that outbreak; however; the circulation of fourteen isolates of *Y. pestis* had been confirmed in the foxes and rodents through their fleas [46]. Nineteen locations from four villages were selected for the flea survey in this focus (Fig 1). Additionally, five locations in the Tello region of Northeastern Tehran Province, central Iran, were chosen as control areas. While no plague outbreaks have been reported, this region has undergone significant environmental changes in recent years [47] (Fig 1). Specimens from Bushehr Province (Borazjan) in Southwestern Iran, which has a warm climate and a history of numerous plague outbreaks, were selected for comparison [41] (Fig 1). Live wooden traps were set adjacent to rodent burrows at dusk and monitored each morning for three consecutive days. Rodents were captured using Sherman live traps a standard technique in small mammal field studies [48]. GPS coordinates were recorded for all sampling points. Sampling was conducted from April to September 2022, coinciding with favorable weather conditions, peak rodent activity, and heightened flea abundance, across the 67 specified locations. After a vehicle collision, a dead red fox found on Akanlu Road in the Shirin Su District, Kabudarahang County, Hamadan Province, was also included in the sampling. The traps were checked on consecutive days, and the trapped specimens were transferred to a flat, shaded, wind- and stress-free area away from human habitation for flea collection. Rodents were restrained by their ears and tails using two long forceps, and fleas were gathered from their bodies by blowing into the hair of the captured animals over a tray of clean water [49]. Fleas were immediately collected from the water and preserved in 70% ethanol within sterile Eppendorf tubes. After flea collection, all rodents were safely released back into their natural habitats.

### Morphological identification of mammals and fleas

The genus and species of the captured rodents were determined based on standard external morphological characteristics, including color, head and body length, ear length, tail length, hind leg length, and dental features [50]. Fleas detached from each host and location were divided into two groups. The first group was employed for morphological identification, while the second group was utilized for molecular analyses, including flea phylogeny and the investigation of *Wolbachia* bacterial infection. The fleas in the first group were mounted with Pouri’s solution on microscope slides. The specimens were carefully examined morphologically under a light microscope (Olympus SZ40, Olympus Corporation, Tokyo, Japan) and identified with the help of a valid taxonomic key for Iranian fleas [51–54]. Key diagnostic morphological features of each specimen were photographed for documentation.

### Molecular survey of fleas and their *Wolbachia* infection

For molecular identification and phylogenetic analysis, one flea per species per sampling location was selected, yielding a total of 58 individual specimens for analysis based on mitochondrial *COII* and nuclear *ITS2* gene sequences. For *Wolbachia* screening, up to eight fleas—comprising four males and four females where possible—were randomly selected per location and flea species, resulting in 102 total specimens screened using three *Wolbachia*-specific markers: *wsp*, *groEL*, and *gatB*. Fleas were chosen randomly from the pool of collected individuals while ensuring representation across host species and geographical sites, to minimize sampling bias and maximize coverage of flea-host-environment interactions. After superficially rinsing the fleas with alcohol 70% and centrifugation, the alcohol-free specimens were ground using a disinfected metal pestle, and total DNA was extracted from the fleas using the Sambio DNA extraction kit (South Korea; Lot: 17F19-16), following the manufacturer’s instructions. PCR amplification of molecular markers targeting flea phylogeny and *Wolbachia* infection was conducted using the primers and thermal conditions listed in Table 1, utilizing a Quants Biotech QB-96 thermal cycler. PCR was carried out in a total volume of 25 μL, which included 1–2 μL of DNA extract (~0.1 μg), 12.5 μL of 2 × Taq DNA Polymerase Master Mix RED (Ampliqon, Denmark), 1 μL of each primer (10 mM), and 8.5-9.5 μL of sterile water. All markers were amplified using conventional PCR, but *wsp* was amplified by semi-nested PCR, as explained before in detail [55–62]. In all PCR assays, both positive and negative controls were included in each run to ensure the reliability and specificity of the amplification. The PCR products were visualized on 1.5% agarose gels stained with GreenViewer and documented using a UV transilluminator. The successful amplicons were purified and subsequently subjected to bidirectional Sanger sequencing by Genomin Company (Tehran, Iran).

**Table 1:** PCR primer sequences and thermal conditions used for the molecular systematics of fleas and determination of their *Wolbachia*/ filarial infection.

Marker	Primer name	Sequence (5′- 3′)	Amplicon size (bp)	Thermal conditions	Reference
*COII*	F-leu R-lys	TCT AAT ATG GCA GAT TAG TGC GAG ACC AGT ACT TGC TTT CAG TC	780 bp	one cycle of 5 min at 95°C, 35 cycles of 30 sec at 94°C, 30 sec at 55°C, and 30 sec at 72°C, with a final step of 5 min at 72°C.	[55]
*ITS2*	ITS2D ITS2R	CAC TGC GCT CGT GGA TCT AT TTT AGG GGG TAG TCT CAC CTG	480 bp	one cycle of 5 min at 94°C, 33 cycles of 30 sec at 94°C, 30 sec at 48°C, and 30 sec at 72°C, with a final step of 7 min at 72°C.	[60]
*wsp*	81F 691R 183F	TGG TCCA ATAAGTGATGAAGAAAC AAAAATTAAACGCTACTCCA AAGGAACCG AAGTTCATG	632 bp	one cycle of 5 min at 95°C, 35 cycles of 1 min at 94°C, 1 min at 55°C, and 1 min at 72°C, with a final step of 7 min at 72°C.	[56]
*groEL*	groEL-F groEL-R	GGTGAGCAGTTRCARSAAGC TARCCRCGRTCAAAYTGCATRCCA	491 bp	one cycle of 3 min at 94°C, 38 cycles of 30 sec at 94°C, 30 sec at 50°C, and 30 sec at 72°C, with a final step of 10 min at 72°C.	[57]
*gatB*	gatB F1 gatB R1	GAKTTAAAYCGYGCAGGBGTT TGGYAAYTCRGGYAAAGATGA	497 bp	one cycle of 3 min at 94°C, 37 cycles of 30 sec at 94°C, 45 sec at 48°C, and 90 sec at 72°C, with a final step of 7 min at 72°C.	[58]
*5.8S-18S*	UNI-1R FIL-1F	CGCAGCTAGCTGCGTTCTTCATCG GTGCTGTAACCATTACCGAAAGG	712-771 bp	**nest-one:** one cycle of 7 min at 94°C, 40 cycles of 20 sec at 94°C, 20 sec at 60°C, and 30 sec at 72°C, with a final step of 10 min at 72°C.	[59]
*18S rDNA-ITS1*	FIL-2F FIL-2R	GGTGAACCTGCGGAAGGATC TGCTTATTAAGTCTACTTAA	286-344 bp	**nest-two:** one cycle of 7 min at 94°C, 35 cycles of 20 sec at 94°C, 20 sec at 50°C, and 20 sec at 72°C, with a final step of 10 min at 72°C.	[59]

### Survey of filarial nematodes associated with fleas

Screening for potential filarial nematode infections in fleas was conducted using PCR with primers designed by Tang et al., [59] targeting the *18S rDNA*-*ITS1* nuclear marker—a region commonly used in filarial diagnostics. The PCR protocol followed the methodology described by Khanzadeh et al [63,64]. Only samples that tested positive for *Wolbachia* supergroup F were included in the screening, as members of this *Wolbachia* supergroup are known endosymbionts of certain filarial nematodes. Primer sequences and PCR conditions are detailed in Table 1.

### Sequence and phylogenetic analyses

A total of 116 nucleotide sequences were analyzed to assess flea genetic diversity and phylogenetic relationships, derived from 58 individual fleas (each sequenced for both mitochondrial *COII* and nuclear *ITS2* markers). For *Wolbachia* screening, 73 sequences were generated from 102 flea specimens screened, representing successful amplifications of one or more of the three *Wolbachia* genes (*wsp*, *groEL*, and *gatB*). These sequences were used independently to investigate the genetic diversity of Iranian flea populations and were combined with sequences retrieved from GenBank to reconstruct phylogenetic trees. The quality of the raw sequence data was initially verified using Chromas 2.6.6 software (Technelysium Pty Ltd., South Brisbane, Australia). BLAST (Basic Local Alignment Search Tool) searches were performed against the GenBank database to compare under-investigated sequences. Multiple sequence alignments were generated using the MUSCLE program embedded in MEGA X software [65]. Basic sequence statistics, including polymorphic and parsimony-informative sites (i.e., alignment positions with at least two nucleotide states, each occurring in two or more sequences), were analyzed using the Sequence Data Explorer tool within the MEGA X software. Inter- and intra-taxa divergences for the *COII* and *ITS2* genes were estimated using the Kimura Two-Parameter (K2P) distance model within MEGA X [65]. Phylogenetic relationships were inferred using the Maximum Likelihood (ML) method with K2P correction models implemented in MEGA X. Bootstrap support (BS) assessed internal node confidence with 1,000 replicates. The sequences acquired in this study have been deposited in the GenBank database.

## Results

### Morphological identification of mammals and fleas

A total of 223 mammals were examined from the endemic plague foci in the Provinces of East Azerbaijan (Sarab), West Azerbaijan (Seyd-abad), Hamadan (Akanlu), and non-endemic northeast of Tehran (Tello) (Fig 1). The animals comprised 222 rodents, especially *Meriones persicus* (n = 210) and *Microtus arvalis* (n = 12), and one fox (*Vulpes vulpes*). All examined mammals harbored fleas, except for six *M*. *arvalis*. Additionally, this study included two previously collected specimens of *X. astia* from *M. persicus* in Bushehr (Borazjan). In total, 1,439 fleas were collected, consisting of 623 males and 816 females. These fleas taxonomically belonged to six species: *X. buxtoni* (n = 1,169), *X. nuttalli* (n = 171), *X. astia* (n = 2), *P. irritans* (n = 54), *N. iranus iranus* (n = 40), and *C. rettigi smiti* (n = 3), representing four genera and three families (Pulicidae, Ceratophyllidae, and Ctenophthalmidae). Table 2 presents detailed information on the flea species, including their abundance, host species, sampling locations, and other relevant data. All collected flea species displayed the expected morphological characteristics for their respective taxa. The diagnostic features of males and females of the six species— *X. buxtoni*, *X. nuttalli*, *X. astia*, *N*. *iranus iranus*, *P*. *irritans*, and *C*. *rettigi smiti*—are illustrated in Figs A-F in S1 Text, which were photographed under a microscope [51–54].

**Table 2:** Data on the fleas under study including their number, taxonomy, sex, host, sampling location, and *Wolbachia* infection rates with different markers.

Sampling sites	Host	Species	Total (M, F)	No. of sequences determined for each marker					No. *Wolbachia*⁺ (F, M)/ Total Tested	*Wolbachia* prevalence (%)
Plague-endemic				*COII*	*ITS2*	*Wsp*	*gatB*	*groEL*		
Hamedan (Akanlu)	*Vulpes vulpes* (n = 1)	*P. irritans*	54 (22,32)	1	1	7	7	8	(6,4) 10/13	77%
	*Meriones persicus* (n = 64)	*X. buxtoni*	481 (224,257)	19	19	7	4	5	(9,7) 16/23	70%
	*Microtus arvalis* (n = 3)	–	0	0	0	0	0	0	0	0
	*Meriones persicus* (n = 9)	*N. iranus iranus*	19 (12,7)	2	2	1	1	–	(1,1) 2/5	40%
West Azerbaijan (Seyed-Abad)	*Meriones persicus* (n = 54)	*X. buxtoni*	317 (145,172)	13	14	3	2	4	(7,6) 13/21	62%
East Azerbaijan (Sarab)	*Meriones persicus* (n = 25)	*X. buxtoni*	172 (62,110)	6	6	2	3	3	(5,4) 9/12	75%
	*Microtus arvalis* (n = 1)	–	0	0	0	0	0	0	0	0
	*Meriones persicus* (n = 17)	*X. nuttalli*	171 (77,94)	7	7	4	2	2	(4,1) 5/9	56%
	*Meriones persicus* (n = 12)	*N. iranus iranus*	17 (4,13)	1	1	1	–	1	(1,0) 1/3	33%
	*Microtus arvalis* (n = 6)	*C. rettigi smiti*	3 (1,2)	1	1	–	–	–	0/1	0
Bushehr (Borazjan)	*Meriones persicus* (n = 1)	*X. astia*	1 (0,1)	1	1	–	–	–	0/1	0
**Non-endemic**										
Tehran (Tello)	*Meriones persicus* (n = 26)	*X. buxtoni*	199 (73,126)	5	5	1	2	3	(6,3) 9/12	75%
	*Microtus arvalis* (n = 2)	–	0	0	0	0	0	0	0	0
	*Meriones persicus* (n = 2)	*N. iranus iranus*	4 (2,2)	1	1	–	–	–	0/2	0
**Total (5)**	**223**	**6**	**1439 (623,816)**	**58**	**58**	**26**	**21**	**26**	**(39,26) 65/102**	**64%**

Abbreviations: M and F stand for male and female specimens.

### Molecular identification of fleas

DNA sequences of six morphologically identified flea species were successfully amplified and sequenced for the *COII* (~780 bp) and *ITS2* (~480 bp) markers from 58 locations. The resulting sequences have been deposited in the GenBank under the accession numbers OR059306-OR059362 and OR939694 for *COII* and also OR081769-OR081825 and PQ164822 for *ITS2* Table A in S1 Text). Analysis of the *COII* sequences revealed 224 (34%) polymorphic sites (i.e., nucleotide positions showing more than one variant among flea samples), of which 151 (23%) were parsimony-informative. There were also 188 (39%) polymorphic sites, with 60 (22%) being parsimony-informative in the *ITS2* gene sequences. Multiple sequence alignments of *COII* of the sequences for *X. buxtoni* revealed nucleotide polymorphisms. In Tello, a T to C substitution was observed at position 167 in some populations. In Hamadan, multiple substitutions were observed: T to A at position 126, T to C at position 179, and T to A at position 309. In Sarab, no nucleotide variations were detected; however, in Bukan, a T to C substitution occurred at position 296 in some individuals. Despite these nucleotide substitutions, no changes in the translated protein sequences were observed at the amino acid level, resulting in 100% intraspecific amino acid similarity among the analyzed *X. buxtoni* populations. Furthermore, in *X. nuttalli* from Sarab, a T to C substitution at position 678 was observed, but this replacement did not result in any amino acid changes, maintaining 100% intraspecific amino acid similarity. In contrast, in *N. iranus iranus* from Sarab, a T to C substitution at position 386 led to an amino acid change from isoleucine to valine. BLAST analysis of *X. buxtoni* sequences revealed 92.31% similarity to *X. gerbilli minax* (KU880673) and 92.01% to *X. conformis conformis* (MF136073). Interestingly, *X. nuttalli* sequences demonstrated 99.56% similarity to *X. conformis conformis* (MF136073). *X. astia* sequences exhibited 92.12% similarity to *X. gerbilli minax* (KU880673) and 92.09% to *X. conformis conformis* (MF136073). *P. irritans* sequences displayed 100% resemblance and query coverage with a similar sequence within the species (LR991748). *C. rettigi smiti* sequences showed the highest similarity (97.14%) to *C. agyrtes* (KM890873). Additionally, *N. iranus iranus* sequences showed 95.64% similarity to *N. laeviceps laeviceps* (PP838812) (Table B in S1 Text). Multiple sequence alignments of the *ITS2* gene of *X. buxtoni* from four locations, *X. nuttalli* from different stations in Sarab, and *N. iranus iranus* from three study locations did not reveal any intraspecific divergence. BLAST analysis of the *ITS2* marker from the six studied flea species revealed a diverse range of results. The sequences of *X*. *buxtoni* exhibited 83.02% similarity to *Synopsyllus fonquernii* and 90.50% similarity to *X. cheopis* (DQ295059). The sequences of *X*. *nuttalli* demonstrated 100% identity with *X. nuttalli* (OR769686), while those of *X*. *astia* exhibited 82.18% similarity to *X. cheopis* (DQ295059). *S*equences of *P*. *irritans* displayed 99.77% similarity to a sequence within the same species (KX982861). The sequences of *N*. *iranus iranus* exhibited 98.17% similarity to *N. barbarus* (LT703445) and 94.69% to *Citellophilus sparsilis* (AY072641). The sequences of *C. rettigi smiti* exhibited 80.95% similarity to both *C. apertus allani* (LR594433) and *C. baeticus boisseauorum* (LR594433) (Table B in S1 Text).

### Genetic distance between studied fleas

**Fig 2:**
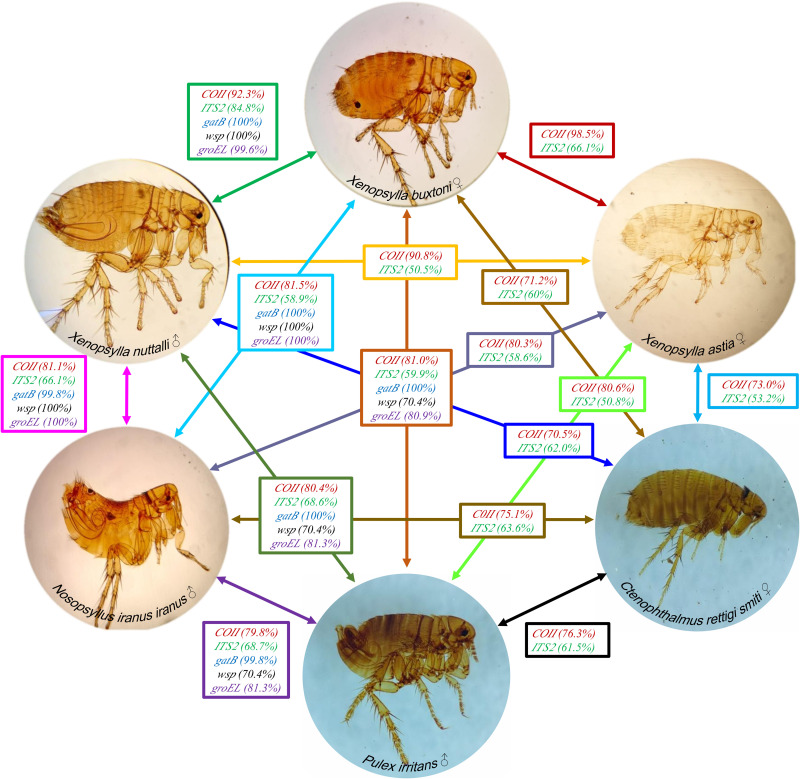
Sequence similarity of six flea species and their infecting *Wolbachia* based on *COII*/*ITS2* and *wsp*/*groEL*/*gatB* markers. Comparisons were highlighted with distinct colors to facilitate the tracing of relationships (Original image by the authors).

Molecular analysis of the *COII* and *ITS2* genes confirmed the morphological distinctiveness of three *Xenopsylla* species: *X. buxtoni*, *X. nuttalli*, and *X. astia*. These findings further supported the separation of *Xenopsylla* species from *P. irritans*. The analysis revealed significant genetic distances between *Xenopsylla* species and representatives of other families, namely *N. iranus iranus* (Ceratophyllidae) and *C. rettigi smiti* (Ctenophthamidae), corroborating the observed morphological differences. *COII* gene sequence similarity among the six species investigated ranged from 70.5% to 98.5%. Notably, while *X. buxtoni* demonstrated the highest *COII* sequence similarity with *X. astia*, its morphological affinities were aligned more closely with *X. nuttalli*. Furthermore, the most significant genetic divergence based on *COII* was observed between *X. nuttalli* and *C. rettigi smiti* (Table C in S1 Text and Fig 2). *ITS2* gene sequence similarity among the six studied flea species ranged from 80.8% to 84.8%. The highest genetic similarity was observed between *X. buxtoni* and *X. nuttalli*, consistent with their close morphological resemblance. Additionally, the highest genetic divergence was observed between *X. astia* and *P. irritans* (Table D in S1 Text and Fig 2).

### Phylogenetic analysis of fleas

**Fig 3:**
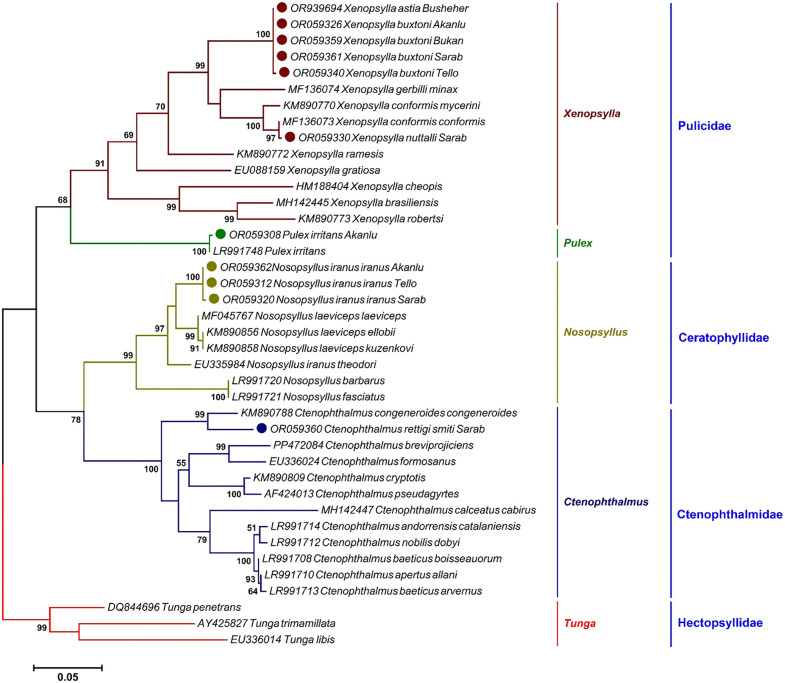
A maximum likelihood tree deduced from 566 bp of the *COII* gene, offering the phylogenetic position of flea taxa under study, alongside other sequences retrieved from the GenBank. The colors indicate the different statuses of the species in each genus. Only eleven representatives of the 58 sequences determined in this study (solid circles) were included in the tree. Three sequences of *Tunga penetrans* (DQ844696), *Tunga trimamilata* (AY425827), and *Tunga libis* (EU336014) were set as outgroups. Bootstrap Support (BS) values (1,000 replicates) are shown at nodes as percentages. Clades with BS ≥ 70% are considered well-supported.

An ML phylogenetic tree was reconstructed based on the *COII* gene, including 30 species representing five genera and four families within the order Siphonaptera. The six flea species studied were accurately placed within their respective lineages, supported by high bootstrap values (Bootstrap Support: 97–100%). Even at the subspecies level, ML analyses strongly supported the monophyly of *N. iranus iranus* and *C. rettigi smiti*, both with a BS > 95%. At the genus level, all four genera—*Pulex*, *Xenopsylla*, *Nosopsyllus*, and *Ctenophthalmus*—were correctly identified as polyphyletic. The family Pulicidae (BS = 68%), which includes *Pulex* and *Xenopsylla* as sister groups, was supported as monophyletic. The family Ceratophyllidae, which contains *Nosopsyllus*, was found to be the sister group of the Ctenophthalmidae clade, which includes *Ctenophthalmus* (Fig 3).

**Fig 4:**
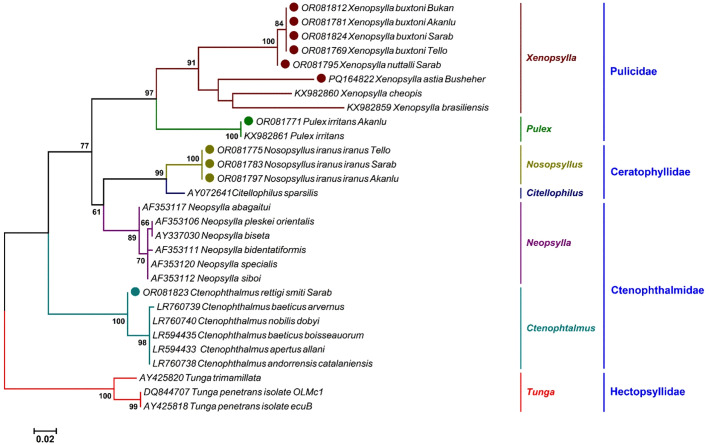
A maximum likelihood tree deduced from 548 bp of the *ITS2* gene presenting the phylogenetic position of flea taxa under study, alongside other sequences retrieved from the GenBank. The colors indicate the different statuses of the species in each genus. Only eleven representatives of the 58 sequences determined in this study (solid circles) were included in the tree. Three sequences of *Tunga penetrans* (DQ844707 and, AY425818) and *Tunga trimamilata* (AY425820) were set as outgroup. Bootstrap Support (BS) values (1,000 replicates) are shown at nodes as percentages. Clades with BS ≥ 70% are considered well-supported.

The phylogenetic analysis based on the *ITS2* gene (Fig 4) included 23 species, representing six genera and four families within the Siphonaptera. At the subspecies level, ML analyses strongly supported the monophyly of *N. iranus iranus* (BS > 95%) and *C. rettigi smiti* (BS > 95%). At the species level, six species formed distinct monophyletic lineages, each with high BS (>95%). At the genus level, all six genera—*Pulex*, *Xenopsylla*, *Nosopsyllus*, *Neopsylla*, *Citellophilu*s, and *Ctenophthalmus*—were strongly confirmed as monophyletic. At the family level, the Pulicidae (BS > 95%), comprising *Pulex* and *Xenopsylla* as sister groups, and the Ceratophyllidae (BS > 95%), comprising *Citellophilus* and *Nosopsyllus*, were both affirmed to be monophyletic. The family Ceratophyllidae, which includes *Nosopsyllus*, is positioned as the sister group to the Ctenophthalmidae clade, which comprises *Ctenophthalmus*.

### Prevalence and supergroup classification of *Wolbachia*, and detection of filarial nematodes in fleas

All flea samples of *X. buxtoni*, *X. nuttalli*, and *N. iranus iranus* that tested positive for *Wolbachia* supergroup F were negative for filarial nematode infection. The PCR method successfully amplified *Wolbachia wsp, groEL*, and *gatB* gene sequences in an average of 64% of the fleas tested, of which 60% were males and 40% were females. The highest infection rate was found in *P. irritans* (77%), and the lowest rates were recorded for *N. iranus iranus* (0%), *C. rettigi smiti* (0%), and *X. astia* (0%). *X. buxtoni* displayed an average infection rate of 69%, with rates ranging from 62% to 70% for specimens from West Azerbaijan and Hamadan Provinces and reaching 75% for specimens from East Azerbaijan and Tehran Provinces. Other flea species showed *Wolbachia* infection rates of 56% for *X. nuttalli* and 33%-40% for *N. iranus iranus* (Table 2). No significant differences in *Wolbachia* infection rates were observed among flea species or between plague-endemic and non-endemic regions (*p* > 0.05). However, infection prevalence was significantly higher in female fleas compared to males (73% vs. 53%; *p* = 0.002) (Table E in S1 Text).

**Fig 5:**
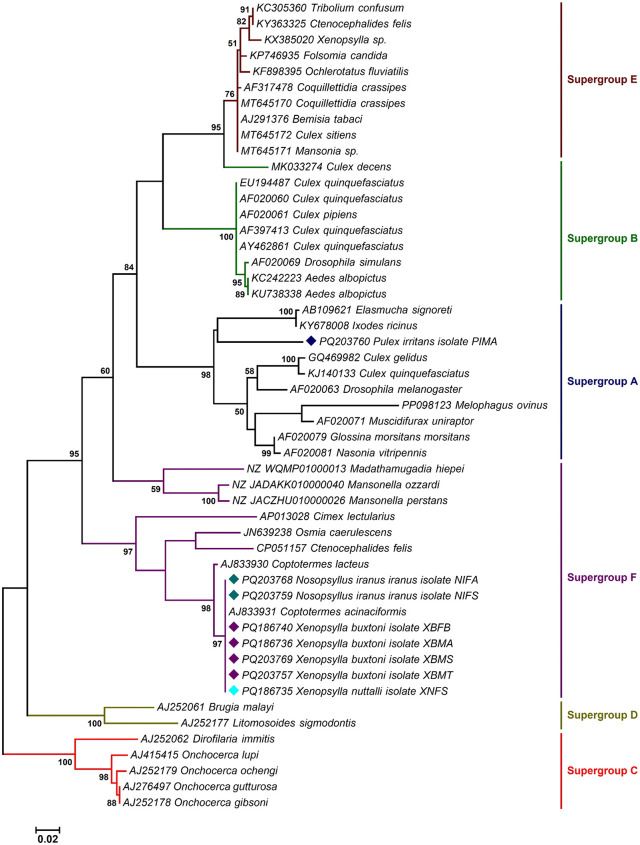
Maximum likelihood phylogenetic tree deduced from 528 bp of the *wsp* gene sequences determining the supergroup position of *Wolbachia* endosymbionts of fleas among other reference sequences retrieved from the GenBank. Isolates identified in this study are marked with colored diamonds. Bootstrap Support (BS) values (1,000 replicates) are shown at nodes as percentages. Clades with BS ≥ 70% are considered well-supported. Bootstrap values lower than 50% are omitted from the branches. The bar indicates substitutions per site. Abbreviations: PIMA: *Pulex irritans* male from the Akanlu; XBFT: *Xenopsylla buxtoni* female from Tello; XNFS: *Xenopsylla nuttalli* female from Sarab; XBMB: *Xenopsylla buxtoni* male from Sarab; XBMA: *Xenopsylla buxtoni* male from Akanlu; XBFB: *Xenopsylla buxtoni* female from Bukan; NIFS: *Nosopsyllus iranus iranus* female from Sarab; NIFA: *Nosopsyllus iranus iranus* female from Akanlu.

**Fig 6:**
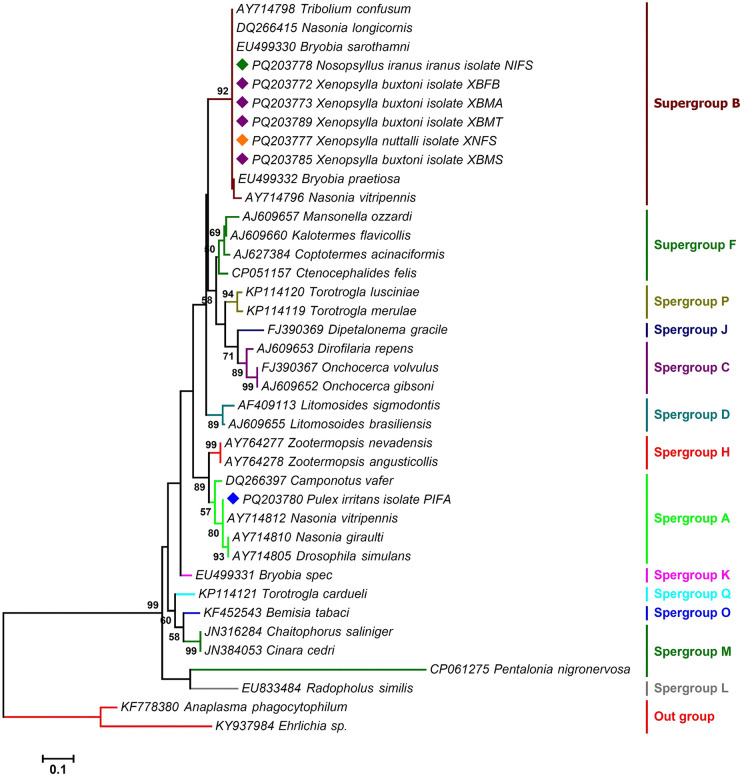
Maximum likelihood phylogenetic tree deduced from 493 bp of the *groEL* gene sequences determining the supergroup position of *Wolbachia* endosymbionts of fleas among other reference sequences retrieved from the GenBank. Isolates identified in this study are marked with colored diamonds. Two sequences of *Anaplasma phagocytophilum* (KF778380, KY4937984) and an *Ehrlichia* sp. (AY425820) were set as outgroups. Bootstrap Support (BS) values (1,000 replicates) are shown at nodes as percentages. Clades with BS ≥ 70% are considered well-supported. Bootstrap values lower than 50% are omitted from the branches. The bar indicates substitutions per site. Abbreviations: XBMT: *Xenopsylla buxtoni* male from Tello; XNFS: *Xenopsylla nuttalli* female from Sarab; XBMS: *Xenopsylla buxtoni* male from Sarab; XBMA: *Xenopsylla buxtoni* male from Akanlu; XBFB: *Xenopsylla buxtoni* female from Bukan; NIFS: *Nosopsyllus iranus iranus* female from Sarab; PIFA: *Pulex irritans* female from Akanlu.

**Fig 7:**
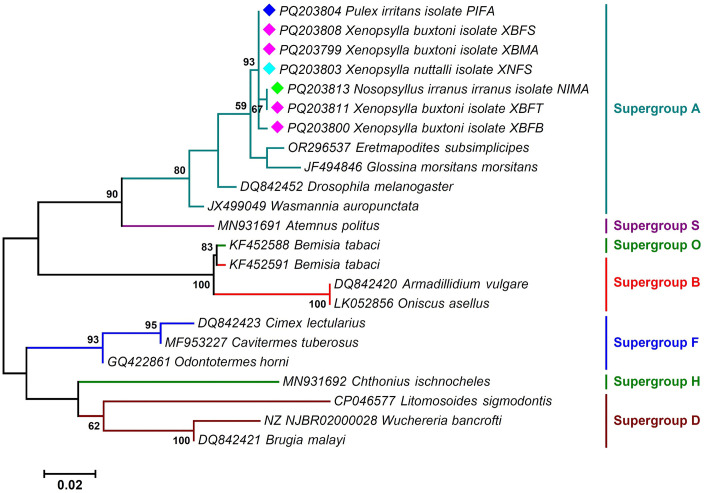
A maximum likelihood phylogenetic tree deduced from 548 bp of the *gatB* gene sequences determining the supergroup position of *Wolbachia* endosymbionts of fleas among other reference sequences retrieved from the GenBank. Isolates identified in this study are marked with colored diamonds. Bootstrap Support (BS) values (1,000 replicates) are shown at nodes as percentages. Clades with BS ≥ 70% are considered well-supported. Bootstrap values lower than 50% are omitted from the branches. The bar indicates substitutions per site. Abbreviations: XBMA: *Xenopsylla buxtoni* male from Akanlu; XNFS: *Xenopsylla nuttalli* female from Sarab; XBFS: *Xenopsylla buxtoni* female from Sarab; PIFA: *Pulex irritans* female from Akanlu; NIMA: *Nosopsyllus iranus iranus* male from Akanlu; XBFT: *Xenopsylla buxtoni* female from Tello; XBFB: *Xenopsylla buxtoni* female from Bukan.

The *Wolbachia* sequences analyzed in this study identified three distinct supergroups: A, B, and F in fleas, each exhibiting varying prevalence rates (Figs 5, 6, 7 and Table A in S1 Text). Supergroup A was the most prevalent, infecting 50% of the specimens. Supergroup F was detected in 33% of the fleas, while supergroup B was found in 27%. Co-infections involving multiple subgroups were also observed. Approximately 14% of the specimens exhibited infections with both supergroups F and B, and 6% were infected with three supergroups A, F, and B. Around 1% of fleas were infected with supergroups F and A, and 7% with supergroups A and B. BLAST analysis of the *wsp* gene sequences showed that the *Wolbachia* infecting the flea species *X. buxtoni*, *X. nuttalli*, and *N. iranus iranus* exhibited 99.81% identity to the *Wolbachia* strains found in the insect *Coptotermes acinaciformis* (AJ833931) and 90.56% identity to the *Wolbachia* strain in *Ctenocephalides felis* (CP051157). The *Wolbachia* sequences from the three mentioned flea species were classified within supergroup F. However, the *wsp* sequence from *P. irritans* was categorized in supergroup A, demonstrating 100% identity with other isolates from different dipteran/lepidopteran insect species (OZ034724, DQ380871, and OZ035007). The presence of multiple *Wolbachia* supergroups in fleas may be attributed to *wsp* gene recombination events, as previously suggested by Baldo et al [66].

Analysis of *groEL* gene sequences showed that the sequences of *Wolbachia* isolated from the flea *X. buxtoni*, *X. nuttalli*, and *N. iranus iranus* exhibited 100% similarity to *Wolbachia* strains found in various insects, including *Tribolium confusum* (AY714798) and *Tholera decimalis* (OZ034777). The *Wolbachia* sequences of these three flea species were classified within supergroup B. However, the *groEL* sequence of the symbiotic *Wolbachia P. irritans* was categorized in supergroup A, showing 100% resemblance to isolates from other insects, such as *Aporus unicolor* and *Chalcis sispes.* Phylogenetic analysis based on *gatB* gene sequences indicated that all *Wolbachia* isolates identified in this study clustered within supergroup A. These sequences were identical to those found in insect hosts, including *Udea olivalis*, *Epagoge grotiana*, and *Nomada hirtipes*.

## Discussion

The current study presents the first comprehensive analysis of flea diversity and the associated *Wolbachia* symbionts in rodent populations across both endemic and non-endemic plague regions in the western half of Iran. By combining morphological and molecular characterization, we identified six distinct flea species, predominantly *X. buxtoni*. Molecular screening revealed widespread *Wolbachia* infections across three supergroups (A, B, and F), with prevalence rates varying by species and ranging from 33% to 77%. A significant difference in infection rates was observed between supergroups A and B (P < 0.02) (Table E in S1 Text).

Phylogenetic analyses based on *ITS2* and *COII* gene markers confirmed the morphological identification of four flea species: *C. rettigi smiti*, *N. iranus iranus*, *P. irritans*, and *X. astia*. The significant genetic divergence observed between *X. buxtoni* and *X. nuttalli*—particularly in both *COII* and *ITS2* sequences—suggests potential cryptic diversity within the conformis group, a morphologically defined subgroup of *Xenopsylla* that includes *X. buxtoni*, *X. nuttalli*, and *X. conformis*. Although traditional taxonomy has grouped these species based on shared morphological traits (e.g., absence of genal and pronotal combs, genital structures), our molecular data reveal deep evolutionary splits that are not reflected in current classifications. This discrepancy has important taxonomic and epidemiological implications: if *X. buxtoni* and *X. nuttalli* represent distinct evolutionary lineages, their vector competence for *Y. pestis* and other pathogens may differ significantly, affecting plague surveillance and risk assessment. We therefore recommend integrative taxonomic studies combining high-resolution morphological analysis, multi-locus molecular markers (e.g., *COI*, *28S*, *EF-1α*), and broader geographic sampling across the Palearctic region to clarify species boundaries and refine the systematic framework of the conformis group.

The molecular identification of *X. buxtoni*, *N. iranus iranus*, *X. nuttalli*, *X. astia*, and *C. rettigi smiti* was hindered primarily by the lack of related species sequences in the GenBank. Nevertheless, this research significantly enriched genomic databases by depositing *COII* and *ITS2* sequences, thereby providing valuable resources for future molecular studies on flea populations.

The phylogenetic study of fleas based on *ITS2* and *COII* gene sequences revealed a strong congruence between molecular and morphological classifications of fleas at the subspecies, species, and genus levels. However, significant genetic distances were observed in some cases, potentially reflecting the absence of homologous sequences in the GenBank for the studied specimens. This observation aligns with previous studies that documented high levels of genetic diversity within specific flea species [12,55].

Integrating molecular analysis with traditional morphological classification has proven invaluable in modern systematic studies, especially when morphological differences are subtle. Historical classifications of *X. buxtoni* and *X. nuttalli*, based on minor morphometric distinctions, established by Hopkins and Rothschild (1953) and Haeselbarth (1966) [67,68], warrant re-evaluation of specific current taxonomic group using contemporary molecular approaches. Our analysis of *ITS2* nucleotide sequences from populations of six flea species revealed unexpected genetic similarity, despite their diverse geographical origins. This finding suggests that the observed patterns are more likely the result of human-mediated introductions rather than long-distance geographical isolation.

MtDNA *COI* and *COII* markers have proven effective for resolving flea phylogenies. *COI* exhibited higher nucleotide diversity, while *COII* demonstrated greater amino acid diversity, it should be noted that this diversity reflects intraspecific variation [69]. Additionally, *COII* revealed the smallest genetic distance between the closely related species *X. buxtoni* and *X. nuttalli*, highlighting its valuable utility in distinguishing closely related taxa.

This study reports, for the first time, the presence of the endosymbiont *Wolbachia* in the fleas *X. buxtoni*, *X. nuttalli*, and *N. iranus iranus*, with a high infection prevalence of 60%. Although *Wolbachia* has previously been detected in other flea species, including *Ctenocephalides canis*, *C. felis*, *Echidnophaga gallinacea*, and *Polygenus gwyni* (25%), infection rates in these species have generally been lower [70]. Prevalence rates for these species range from 7% in *C. canis* to 24% in *E. gallinacea* and 21% in *C. felis*, which are significantly lower than the prevalence observed in *X. buxtoni*, *X. nuttalli*, *N. iranus iranus*, and *P. irritans*. The high prevalence detected in the present study corroborates findings from other flea species, such as *T. penetrans*, *P. simulans* (94%), *Archaeopsylla erinaceid* (95%), and *Orchopeas howardi* (80%), which exhibit infection rates ranging from 80% to 100% [70–72]. Consistent with previous research, we observed a higher prevalence of *Wolbachia* in female than male fleas [73,74]. The female-biased *Wolbachia* infection pattern observed in this study aligns with its canonical mode of maternal transmission, whereby the bacterium is passed through the egg cytoplasm from mother to offspring. Because males represent an evolutionary dead end for *Wolbachia*, high infection prevalence in females is essential for its persistence in host populations. This sex bias may further reinforce *Wolbachia*-induced reproductive manipulations—such as cytoplasmic incompatibility—that enhance the relative fitness of infected females. Altogether, the elevated *Wolbachia* prevalence in female fleas likely results from a combination of strict maternal inheritance and sex-specific host–symbiont interactions that jointly drive the maintenance and spread of the endosymbiont in natural flea populations [75,76]. The high similarity of *Wolbachia* strains identified in *X. buxtoni*, *X. nuttalli*, and *N. iranus iranus* (Fig 2) may reflect host shifts and suggest the potential for horizontal transmission of *Wolbachia* among these species [77]. In male fleas, *Wolbachia* may influence sexual differentiation and development by modulating DNA methylation patterns of sex-determining genes, similar to mechanisms observed in other insect species [78–80]. While *Wolbachia* is known to confer certain benefits to female hosts, its overall role in arthropods is often parasitic, potentially affecting host fitness by altering iron homeostasis and enhancing immunity against viral infections and onchocercoid nematodes [81–83]. Future studies will investigate the role of the *Wolbachia* lineages found in the studied fleas and their interactions with the pathogens they transmit.

The present study, along with others, demonstrates that fleas are frequently infected with multiple *Wolbachia* types [84]. Earlier sequence analyses of *C. felis* (strain wCte) had placed it in supergroups B or F, based on limited genetic data [85,86]. However, recent genome-based studies have provided a more refined understanding of *Wolbachia* diversity in *C. felis*. Notably, strains wCfeT and wCfeJ have been shown to form paraphyletic clades associated with supergroups A and B, exhibiting close relationships with supergroup C [84]. A recent study by Beliavskaia et al. further clarified this complexity, revealing that wCfeT belongs to supergroup I and wCfeJ to supergroup V, highlighting the evolutionary diversity of *Wolbachia* in fleas [87]. In the present study, phylogenetic analyses of *Wolbachia* strains from four flea species—*X. buxtoni*, *X. nuttalli*, *N. iranus iranus*, and *P. irritans*—revealed conflicting supergroup assignments depending on the genetic marker used. Strains clustered primarily in supergroups A and B based on *groEL*, whereas *wsp* placed them in A and F. In contrast, all strains resolved exclusively within supergroup A in the *gatB*-based phylogeny. These inconsistencies likely stem from differences in evolutionary rates among loci, recombination in the *wsp* gene, horizontal transfer of *Wolbachia* strains, or potential primer bias affecting amplification efficiency across divergent lineages [56].

The conflicting supergroup assignments obtained from *wsp*, *groEL*, and *gatB* highlight the limitations of single-marker approaches and underscore that these findings require validation through more robust genomic methods, such as MLST or whole-genome sequencing, to resolve the true phylogenetic placement and potential recombination events among *Wolbachia* lineages.

The detection of *Wolbachia* strains belonging to supergroups A, B, and F across multiple flea species indicates considerable symbiont diversity [47]. Although all samples positive for supergroup F were negative for filarial nematode infection (see Results), the co-occurrence of multiple supergroups within individual fleas suggests potential for intra-host interactions, horizontal transfer, or recombination among lineages [88–89]. Further genomic studies are warranted to investigate the presence of cytoplasmic incompatibility loci and to assess whether recombination—particularly in hypervariable genes such as *wsp*—has contributed to the observed phylogenetic incongruence. Despite marker-specific inconsistencies, our findings provide robust evidence of extensive *Wolbachia* diversity in Iranian flea populations and underscore the need for whole-genome or multi-locus approaches to resolve the evolutionary relationships among these strains.

The broad host range and high vector competence of *X*. *buxtoni*, *X. nuttalli*, *N. iranus iranus*, and *P. irritans* [39] in transmitting numerous significant pathogens—such as *Y. pestis*, *R. felis* and *B. henselae* [42–44,46,90]—underscore the urgent need for effective control strategies targeting these flea species. Vector control based on specific *Wolbachia* strains offers a promising biological approach, exploiting the ability of the bacteria to induce reproductive manipulations and maternal transmission in arthropods to reduce vector populations [91]. This potential has not yet been experimentally validated in fleas. The influence of endosymbionts on critical factors affecting vectorial capacity, such as longevity and reproductive success, may also be modulated by environmental conditions [26,92]. Future studies should experimentally assess the impact of specific *Wolbachia* strains on flea fitness, reproduction, and pathogen transmission to evaluate their feasibility as biocontrol agents.

The recently identified *w*CfeJ strain of *C. felis* contains an antitoxin operon similar to the *w*Pip *cinAB* operon, which has been shown to induce CI in flies. The presence of *Wolbachia* supergroup F in the fleas analyzed in this study, which is closely related to this strain, suggests the potential for discovering similar genomic regions in these fleas, further supporting the role of *Wolbachia* in reproductive interference [84]. This observation, although not investigated in our study, suggests the theoretical possibility that *Wolbachia*-mediated reproductive manipulation could influence flea population dynamics; however, experimental studies are needed to evaluate its feasibility for vector control. Furthermore, the *Wolbachia* strain identified in this study, belonging to supergroup F, is phylogenetically closely related to *Wolbachia* strains found in termites (*Coptotermes*). Although supergroup F remains relatively understudied in termites, several key biological functions have been attributed to *Wolbachia* in termite symbioses. These functions include the induction of CI, protection against pathogens, and enhancement of reproductive success, wood digestion, and immune function [93].

The high sequence similarity and close phylogenetic relationships observed between *Wolbachia* supergroups A and B in various flea species, along with their similarity to strains found in bees, butterflies, and flies, suggest that *Wolbachia* endosymbionts are transmitted not only vertically from mother to offspring but also horizontally [88,93]. Furthermore, studies have demonstrated that *Wolbachia* can be transmitted across species through host switching, a phenomenon that is more common than previously thought for supergroups A and B. Host switching has even been observed between distantly related species, further highlighting the adaptability and widespread nature of *Wolbachia* transmission [33].

A comparison between plague-endemic and non-endemic areas as a control region revealed no significant differences in flea fauna composition and the presence of *Wolbachia* infection. *X. buxtoni* was the dominant flea species in both regions, suggesting that its mere presence may not be a decisive in determining plague endemicity. Molecular analysis using *ITS2* and *COII* gene markers confirmed no intraspecific genetic variation between *X. buxtoni* and *N. iranus iranus* across both areas. The prevalence of *Wolbachia* infection was similar between plague-endemic and non-endemic regions, with supergroups A, B, and F all identified in *X. buxtoni*. In contrast, *N. iranus iranus* from the non-endemic region showed no evidence of *Wolbachia* infection; however, this observation is based on only two specimens. Given the limited sample size, no definitive conclusions can be drawn regarding the absence of *Wolbachia* in this flea species or its potential role in regional transmission dynamics. Further research is needed to explore the role of *Wolbachia* in flea ecology and its interactions with other pathogens. These findings underscore the complexity of plague ecology and suggest that factors beyond flea species composition and *Wolbachia* infection may contribute to plague persistence in endemic regions.

As previously noted, the risk of human plague is highest where natural plague foci intersect with human populations, and flea-associated microbiota are often biased toward pathogenic bacteria [47]. While our study identified diverse *Wolbachia* lineages in fleas, further research is needed to assess their potential relevance to vector control or disease mitigation.

## Conclusion

By identifying *X. buxtoni* as the dominant flea species and detecting *Wolbachia* infections across three supergroups, the current study highlights the intricate host-parasite interactions within different ecosystems. Phylogenetic analyses revealed significant genetic diversity both within and between flea species, emphasizing the need for further studies to refine flea taxonomy, particularly within the conformis group, and to better understand the evolutionary forces shaping these populations. The study highlights the importance of integrating both morphological and molecular approaches to achieve a comprehensive understanding of flea diversity. The high prevalence of *Wolbachia* in plague-carrying fleas likely reflects their critical role in flea biology, host-parasite dynamics, and the transmission of flea-borne diseases. These findings also lay the groundwork for future studies investigating the influence of *Wolbachia* on flea bioecology and its potential applications in controlling flea populations and the pathogens they transmit.

## Supporting information

S1 Text **Fig A.** Specific identification characters of *Xenopsylla buxtoni*. A: Female with a smooth anterior margin of the head and without both genal and pronotal combs. B: Male with a shallow occipital groove and a straight ventral outline. C: Mesopleuron of a female with a pleural rod and the suture that separates the sternum from the episternum of the metathorax. D: The oval bulga of spermathecal characterized by a slightly swollen, dark hilla at the base. E: The penis-plate uniformly widened to an obtuse apex, lacking a produced dorso-apical angle. F: Abrupt narrowing of the hind coxa below the middle of the posterior margin. G: The hind tarsus with only one apical bristle on segment II that extends beyond segment IV. H: The male’s fore tarsal segment V with two sub-apical plantar spiniform bristles. I: The hind tibia in males with tiny, fine bristles between the fourth and fifth pair of dorsal bristles (Original image by the authors). **Fig B.** Specific identification characters of *Xenopsylla nuttalli*. A: Female with a smooth anterior margin of the head and without both genal and pronotal combs. B: Male with a shallow occipital groove and a straight ventral outline. C: Mesopleuron of a female with a pleural rod and the suture that separates the sternum from the episternum of the metathorax. D: The oval bulga of spermatheca characterized by a slightly swollen, dark hilla at the base. E: The penis-plate uniformly widened to an obtuse apex, without a produced dorso-apical angle. F: Abrupt narrowing of the hind coxa below the middle of the posterior margin. G: The hind tarsus with two apical bristles on segment II extending beyond segment IV. H: The male’s fore tarsal segment V with two sub-apical plantar spiniform bristles. (I) The hind tibia in males with tiny, fine bristles between the fourth and fifth pair of dorsal bristles (Original image by the authors). **Fig C.** Specific identification characters of *Xenopsylla astia*. A: Female with a smooth anterior margin of the head and without both genal and pronotal combs. B: Male with a deep occipital groove and undulate ventral outline. C: Mesopleuron of a female with a pleural rod and the suture that separates the sternum from the episternum of the metathorax. D: The subspherical bulga of spermatheca characterized by a swollen hilla at the base twice as wide as the bulga. E: The penis-plate notably broad, with a pronounced convexity at apex, and pre-apically undulating ventral outline. F: Abrupt narrowing of the hind coxa below the middle of the posterior margin. G: Sternum IV-VII of the abdomen often with more than 13 bristles on the two sides together and an outer surface of it. VIII rarely with fewer than 30 including marginal row. H: Sternum VIII typically with 14–27 bristles on each side, the antepygidial bristle located submarginally, flanked by smaller bristles. I: The male’s fore tarsal segment V with three sub-apical plantar spiniform bristles (Original image by the authors). **Fig D.** Specific identification characters of *Nosopsyllus iranus iranus*. A: Female with a smooth anterior margin of the head and genal comb absent, while a pronotal comb always present, typically containing less than 24 spines on the two sides together. B: Fracticipit, occipital groove shallow and a straight ventral outline. C: The apical bristle of segment II of the hind tarsus reaching beyond segment IV. D: Spermatheca with a globular bulga, and shorter than the hilla. E: The posterior margin of the fixed process slopes forward in the middle, movable process of clasper slender with triangular apex, acetabular setas arising above the point of articulation of movable process. F: The male’s fore tarsal segment V with two sub-apical plantar spiniform bristles. G: The posterior margin of the Sternum VII in the female with two protrusions and a distinct depression. H: The penis plate resembles a dagger, with a sharp tip. I: The apical arm of sternum IX in males with a long beak-shaped projection (Original image by the authors). **Fig E.** Specific identification characters of *Pulex irritans*. A: The head with a smooth anterior margin without a tubercle. B: Club of antenna asymmetrical, the anterior segments foliaceous and leaning backwards. C: Genal, pre-ocular, and post-antennal setae of the head. D: Sternite VII of females with a sinus and 4/5 setae on each side. E: Clasper with P1 very large and completely covering P2 and P3, ovoid but with the posterio-distal angle nearly straight; P2 and P3 about three-quarters length of P1. F: The penis-plate resembles a dagger with a sharp tip. G: The spermatheca with a subglobular bulga and a hilla longer than the bulga. H: Dorsal aedeagal sclerite (das) of males long and slender. I: The ventral margin of the hind coxa with a row of 6–20 spiniform setae near the apex, often irregular and forming a patch (Original image by the authors). **Fig F.** Specific identification characters of *Ctenophthalmus rettigi smiti*. A: The pronotal comb with 18 spines, and the labial palp without a curved apical bristle. B: Genal comb horizontal, with three peg-like spines all of visible in side view and directed obliquely backward. Frons with two rows of bristles. C: The shallow occipital groove and the straight ventral outline in males. The eye is greatly reduced but present in males. D: Fixed process undivided; movable process elongated triangular, with about 10 sensilla along anterior margin, aedeagus with preapical dorsal expansion. E: The apical bristle of segment II of the hind tarsus reaching beyond segment IV. F: The antennal club unmodified and consists of nine segments. G: External coxal ridge present. H: Tooth at apex of hind tibia generally pointed. I: Fracticipit and occiput with three rows of bristles (Original image by the authors). **Table A**. Data on the fleas and associated *Wolbachia* including abundance, species identification, sampling locations, and GenBank accession numbers for five *COII*, *ITS2*, *wsp*, *groEL*, *gatB* markers. **Table B**. Megablast results for the sequences obtained in this study and the reference species identified from GenBank with the highest identity and minimum genetic distance. **Table C**. Genetic distances ± standard deviations (SD) among studied flea species based on 660–732 bp of *COII* gene sequences. **Table D**. Genetic distances ± standard deviations (SD) among studied flea species based on 333–486 bp of *ITS2* gene sequences. **Table E**. Comparison of *Wolbachia* infection prevalence among flea populations by sex, geographic location, and bacterial supergroup.
(DOCX)
