# Supplementary material for: Xenopsylla buxtoni fleas as a dominant species harboring multiple infections of Wolbachia lineages in the ancient plague epicenters of Iran
Source: PLoS Negl Trop Dis. 2026 Feb 13;20(2):e0013890. doi: 10.1371/journal.pntd.0013890 (PMC12904580; doi:10.1371/journal.pntd.0013890)
Supplement: S1 Text — Fig A. Specific identification characters of Xenopsylla buxtoni. A: Female with a smooth anterior margin of the head and without both genal and pronotal combs. B: Male with a shallow occipital groove and a straight ventral outline. C: Mesopleuron of a female with a pleural rod and the suture that separates the sternum from the episternum of the metathorax. D: The oval bulga of spermathecal characterized by a slightly swollen, dark hilla at the base. E: The penis-plate uniformly widened to an obtuse apex, lacking a produced dorso-apical angle. F: Abrupt narrowing of the hind coxa below the middle of the posterior margin. G: The hind tarsus with only one apical bristle on segment II that extends beyond segment IV. H: The male’s fore tarsal segment V with two sub-apical plantar spiniform bristles. I: The hind tibia in males with tiny, fine bristles between the fourth and fifth pair of dorsal bristles (Original image by the authors). Fig B. Specific identification characters of Xenopsylla nuttalli. A: Female with a smooth anterior margin of the head and without both genal and pronotal combs. B: Male with a shallow occipital groove and a straight ventral outline. C: Mesopleuron of a female with a pleural rod and the suture that separates the sternum from the episternum of the metathorax. D: The oval bulga of spermatheca characterized by a slightly swollen, dark hilla at the base. E: The penis-plate uniformly widened to an obtuse apex, without a produced dorso-apical angle. F: Abrupt narrowing of the hind coxa below the middle of the posterior margin. G: The hind tarsus with two apical bristles on segment II extending beyond segment IV. H: The male’s fore tarsal segment V with two sub-apical plantar spiniform bristles. (I) The hind tibia in males with tiny, fine bristles between the fourth and fifth pair of dorsal bristles (Original image by the authors). Fig C. Specific identification characters of Xenopsylla astia. A: Female with a smooth anterior margin of the head and [file pntd.0013890.s001.docx]

**Table A**. Data on the fleas and associated *Wolbachia* including abundance, species identification, sampling locations, and GenBank accession numbers for five *COII*, *ITS2*, *wsp*, *groEL*, *gatB* markers.

| Location | Longitude, latitude | Species | Sex and Sample ID | Total (M/F) | Accession number | | Accession number (supergroup assigned | | |
| --- | --- | --- | --- | --- | --- | --- | --- | --- | --- |
|  |  |  |  |  | *COII* | *ITS2* | *wsp* | *gatB* | *groEL* |
| Hamedan, Akanlu: Qeytar Mezruk | X:246019.345  Y:3952635.321 | *Xenopsylla buxtoni* | XBMA1 | 9 (4,5) | OR059309 | OR081772 | PQ186736 (F) | PQ203799 (A) | PQ203773 (B) |
| Hamedan, Akanlu: Kallik | X:234851.179  Y:3937096.595 | *Xenopsylla buxtoni* | XBMA2 | 74 (21,53) | OR059313 | OR081776 | PQ186737 (F) | --- | PQ203774 (B) |
| Hamedan, Akanlu: Qeytar Mezruk | X: 226010.006  Y:3944767.661 | *Xenopsylla buxtoni* | XBMA3 | 35 (16,19) | OR059314 | OR081777 | PQ186738 (F) | PQ203801 (A) | --- |
| Hamedan, Akanlu: Qara Gol | X:237619.058  Y:3948890.92 | --- | --- | 0 (0,0) | --- | --- | --- | --- | --- |
| Hamedan, Akanlu: Bashqurtaran | X:238272.572  Y:3948510.77 | *Xenopsylla buxtoni* | XBMA4 | 4 (2,2) | OR059315 | OR081778 | PQ186743 (F) | --- | --- |
| Hamedan, Akanlu: Akanlu | X:235582.616  Y:3938978.168 | *Pulex irritans.* | PIMA  PIFA | 54 (22,32) | OR059308 | OR081771 | PQ203760  -66 (A) | PQ203804  PQ203806-07  PQ203810  PQ203815  PQ203818-19 (A) | PQ203780-84  PQ203788  PQ203790  PQ203795 (A) |
| Hamedan, Akanlu: Bashqurtaran | X:236990.806  Y:3948761.417 | *Xenopsylla buxtoni* | XBFA2 | 9 (4,5) | OR059316 | OR081779 | PQ186744 (F) | PQ203817 (A) | --- |
| Hamedan, Akanlu: Bashqurtaran | X: 237006.424  Y:3947416.009 | *Xenopsylla buxtoni* | XBFA3 | 14 (7,7) | OR059317 | OR081780 | PQ203767 (F) | --- | --- |
| Hamedan, Akanlu: Kallik | X:234315.573  Y:3939015.808 | *Xenopsylla buxtoni* | XBFA1 | 27 (16,11) | OR059318 | OR081781 | PQ186739 (F) | --- | PQ203776 (B) |
| Hamedan, Akanlu: Kallik | X:236852.221  Y:3936299.836 | *Xenopsylla buxtoni* | XBFA | 5 (2,3) | OR059319 | OR081782 | --- | --- | --- |
| Hamedan, Akanlu: Yekeh Chalab Klik | X:236825.672  Y:3936340.103 | *Xenopsylla buxtoni* | XBFA4 | 60 (29,31) | OR059321 | OR081784 | --- | --- | PQ203791 (B) |
| Hamedan, Akanlu: Kallik | X:236682.151  Y:3936346.799 | *Xenopsylla buxtoni* | XBMA | 26 (16,10) | OR059322 | OR081785 | --- | --- | --- |
| Hamedan, Akanlu: Pir Badam | X:226978.435  Y:3945925.674 | *Xenopsylla buxtoni* | XBFA5 | 4 (2,2) | OR059323 | OR081786 | --- | PQ203805 (A) | PQ203792 (B) |
| Hamedan, Akanlu: Kohneh Hesar | X:233574.299  Y:3937396.818 | *Xenopsylla buxtoni* | XBFA | 25 (11,14) | OR059324 | OR081787 | --- | --- | --- |
| Hamedan, Akanlu: Bashqurtaran | X:238565.079  Y:3948354.083 | *Xenopsylla buxtoni* | XBMA | 24(12,12) | OR059325 | OR081788 | --- | --- | --- |
| Hamedan, Akanlu: Bashqurtaran | X:236467.097  Y:3946466.425 | *Xenopsylla buxtoni* | XBFA | 20 (8,12) | OR059326 | OR081789 | --- | --- | --- |
| Hamedan, Akanlu: Kohneh Hesar | X:229769.675  Y:3938402.721 | *Xenopsylla buxtoni* | --- | 1 (1,0) | --- | --- | --- | --- | --- |
| Hamedan, Akanlu: Yekeh Chalab Klik | X:234315.573  Y:3939015.808 | *Xenopsylla buxtoni* | XBMA | 7 (4,3) | OR059327 | OR081790 | --- | --- | --- |
| Hamedan, Akanlu: Bashqurtaran | X:237437.095  Y:3947588.371 | *Xenopsylla buxtoni* | --- | 0 (0,0) | --- | --- | --- | --- | --- |
| Hamedan, Akanlu: Bashqurtaran | X:236200.382  Y:3946135.02 | *Xenopsylla buxtoni* | XBFA | 10 (3,7) | OR081798 | OR059335 | --- | --- | --- |
| Hamedan, Akanlu: Bashqurtaran | X:234762.338  Y:3946054.364 | *Xenopsylla buxtoni* | --- | 2 (2,0) | --- | --- | --- | --- | --- |
| Hamedan, Akanlu: Bashqurtaran | X:236988.381  Y:3948764.57 | --- | --- | 0 (0,0) | --- | --- | --- | --- | --- |
| Hamedan, Akanlu: Bashqurtaran | X:237041.776  Y:3948611.849 | --- | --- | 0 (0,0) | --- | --- | --- | --- | --- |
| Hamedan, Akanlu: Bashqurtaran | X:236958.32  Y:3948429.235 | *Xenopsylla buxtoni* | XBFA | 67 (38,29) | OR059336 | OR081799 | --- | --- | --- |
| Hamedan, Akanlu: Dali Chu | X:228040.351  Y:3944017.172 | *Xenopsylla buxtoni* | XBMA | 28 (15,13 | OR059337 | OR081800 | --- | --- | --- |
| Hamedan, Akanlu: Dali Chu | X:228065.871  Y:3944027.805 | *Xenopsylla buxtoni*  *Nosopsyllus iranus iranus* | NIFA1 | 42 (19,23) | OR059353  R059362 | OR081816  R081825 | PQ203768 (F) | PQ203800 (A) | --- |
| Hamedan, Akanlu: Dali Chu | X:233341.276  Y:3950070.11 | *Nosopsyllus iranus iranus* | NIMA1 | 7 (4,3) | OR059334 | OR081797 | --- | PQ203813 (A) | --- |
| Hamedan, Akanlu: Chopoqly | X:233220.132  Y:3950227.999 | --- | --- | 0 (0,0) | --- | --- | --- | --- | --- |
| Sum | 28 | 3 | 28 | 554 (258,296) | 22 | 22 | 15 | 12 | 13 |
| West Azerbaijan, Seyed-Abad: A | X:597533.791  Y:4036973.256 | *Xenopsylla buxtoni* | XBFB1 | 4 (2,2) | OR059343 | OR081815 | PQ186740 (F) | PQ203800 (A) | PQ203772 (B) |
| West Azerbaijan, Seyed-Abad: B | X:597853.689  Y:4036637.874 | *Xenopsylla buxtoni* | XBFB | 35 (15,20) | OR059344 | OR081814 | --- | --- | --- |
| West Azerbaijan, Seyed-Abad: C | X:598240.075  Y:4036367.971 | *Xenopsylla buxtoni* | XBMB1 | 7 (3,4) | OR059345 | OR081813 | PQ186741 (F) | PQ203802 (A) | --- |
| West Azerbaijan, Seyed-Abad: D | X:596885.64  Y:4035511.363 | *Xenopsylla buxtoni* | XBMB2 | 38 (16,22) | OR059346 | OR081812 | --- | --- | PQ203775 (B) |
| West Azerbaijan, Seyed-Abad: E | X:597423.958  Y:4036780.932 | *Xenopsylla buxtoni* | XBFB | 14 (6,8) | OR059347 | OR081811 | --- | --- | --- |
| West Azerbaijan, Seyed-Abad: F | X:597627.269  Y:4036410.345 | *Xenopsylla buxtoni* | XBMB | 39 (17,22) | OR059348 | OR081810 | --- | --- | --- |
| West Azerbaijan, Shahrikand: A | X:597461.899  Y:4036947.77 | *Xenopsylla buxtoni* | --- | 2 (1,1) | --- | --- | --- | --- | --- |
| West Azerbaijan, Shahrikand: B | X:598125.117  Y:4035312.699 | *Xenopsylla buxtoni* | XBMB3 | 30 (12,18) | OR059349 | OR081809 | --- | --- | PQ203779 (B) |
| West Azerbaijan, Shahrikand: C | X:598084.477  Y:4036699.049 | *Xenopsylla buxtoni* | XBFB | 28 (14,14) | OR059350 | OR081808 | --- | --- | --- |
| West Azerbaijan, Shahrikand: D | X:598105.399  Y:4036391.115 | *Xenopsylla buxtoni* | XBMB | 29 (14,15) | OR059351 | OR081807 | --- | --- | --- |
| West Azerbaijan, Shahrikand: E | X:597348.949  Y:4036810.916 | *Xenopsylla buxtoni* | XBFB | 62 (34,28) | OR059352 | OR081806 | --- | --- | --- |
| West Azerbaijan, Seyed-Abad: G | X:597509.6  Y:4036911.349 | *Xenopsylla buxtoni* | XBMB4 | 3 (1,2) | OR059356 | OR081819 | PQ186742 (F) | --- | --- |
| West Azerbaijan, Seyed-Abad: H | X:597878.228  Y:4036668.956 | *Xenopsylla buxtoni* | XBFB | 8 (3,5) | OR059357 | OR081820 | --- | --- | --- |
| West Azerbaijan, Seyed-Abad: I | X:598483.777  Y:4037261.383 | *Xenopsylla buxtoni* | XBMB | 7 (3,4) | OR059358 | OR081821 | --- | --- | --- |
| West Azerbaijan, Seyed-Abad: J | X:598331.374  Y:4037530.858 | *Xenopsylla buxtoni* | XBFB2 | 11 (4,7) | OR059359 | OR081822 | --- | --- | PQ203787 (B) |
| Sum | 15 | 1 | 15 | 317 (145,172) | 14 | 14 | 3 | 2 | 4 |
| East Azerbaijan, Sarab: Razliq | X:723051.738  Y:4213435.56 | *Xenopsylla buxtoni* | XBFS | 1 (0,1) | --- | --- | --- | --- | --- |
| East Azerbaijan, Sarab: Khatunabad | X:711340.18  Y:4202438.117 | *Xenopsylla nuttalli* | XNFS1 | 5 (3,2) | OR059328 | OR081791 | PQ186735 (F) | PQ203803 (A) | PQ203777 (B) |
| East Azerbaijan, Sarab: Khatunabad | X:711360.507  Y:4202123.989 | *Xenopsylla nuttalli* | XNFS2 | 12 (8,4) | OR059329 | OR081792 | PQ203758 (F) | --- | PQ203797 (B) |
| East Azerbaijan, Sarab: Cherlu | X:711316.644  Y:4202119.776 | *Xenopsylla buxtoni* | XBMS1 | 61 (26,35) | OR059342 | OR081805 | PQ203769 (F) | PQ203809 (A) | PQ203785 (B) |
| East Azerbaijan, Sarab: Idehlu | X:711212.545  Y:4202083.147 | *Ctenophthalmus rettigi smiti* | XBMS | 3 (1,2) | OR059360 | OR081823 | --- | --- | --- |
| East Azerbaijan, Sarab: Idehlu | X:711383.423  Y:4200101.012 | *Xenopsylla buxtoni* | XBMS | 2 (0,2) | --- | --- | --- | --- | --- |
| East Azerbaijan, Sarab: Idehlu | X:711340.18  Y:4202438.117 | *Xenopsylla nuttalli* | XNFS4 | 4 (0,4) | OR059330 | OR081793 | PQ271629 (F) | --- | --- |
| East Azerbaijan-Idehlu | X:711026.901  Y:4202840.29 | *Xenopsylla buxtoni* | XBMS2 | 72 (29,43) | OR059361 | OR081824 |  | - | PQ203786 (B) |
| East Azerbaijan, Sarab: Khatunabad | X:721136.369  Y:4210828.997 | *Xenopsylla nuttalli* | XNMS1 | 12 (6,6) | OR059332 | OR081795 | --- | PQ203814 (A) | --- |
| East Azerbaijan, Sarab: Khatunabad | X:711577.412  Y:4200726.059 | *Xenopsylla nuttalli* | XNFS | 12 (5,7) | OR059333 | OR081796 | --- | --- | --- |
| East Azerbaijan, Sarab: Khatunabad | X:711577.412  Y:4200726.059 | *Xenopsylla buxtoni* | XBMS | 1 (0,1) | --- | --- | --- | --- | --- |
| East Azerbaijan, Sarab: Idehlu | X:753470.07  Y:3985539.411 | --- | --- | 0 (0,0) | --- | --- | --- | --- | --- |
| East Azerbaijan, Sarab: Idehlu | X:756612.538  Y:3972076.337 | *Xenopsylla buxtoni* | XBFS1 | 54 (17,37) | OR059354 | OR081817 | PQ203770 (F) | PQ203808 (B) | PQ203796 (A) |
| East Azerbaijan, Sarab: Idehlu | X:748953.131  Y:4209858.374 | *Xenopsylla nuttalli* | XNFS3 | 7 (0,7) | OR059338 | OR081801 | PQ203771 (F) |  |  |
| East Azerbaijan, Sarab: Idehlu | X:748985.542  Y:4208795.364 | *Xenopsylla buxtoni* | XBMS | 72 (26,46) | OR059341 | OR081804 | --- | --- | --- |
| East Azerbaijan, Sarab: Cherlu | X:710780.925  Y:4200440.234 | *Xenopsylla buxtoni* | XBFS | 26 (12,14) | OR059339 | OR081802 | --- | --- | --- |
| East Azerbaijan, Sarab: Cherlu | X:712947.266  Y:4199913.267 | *Xenopsylla buxtoni* | XBMS | 1 (0,1) | --- | --- | --- | --- | --- |
| East Azerbaijan, Sarab: Cherlu | X:710831.628  Y:4199324.888 | *Xenopsylla buxtoni* | XBFS2 | 10 (4,6) | OR059331 | OR081794 | --- | PQ203812 (B) |  |
| East Azerbaijan, Sarab: Idehlu | X:742354.297  Y:4204269.917 | *Xenopsylla nuttalli*  *Nosopsyllus iranus iranus* | NIFS1 | 8 (4,4) | OR059355  OR059320 | OR081818  OR081783 | PQ203759 (F) | --- | PQ203778 (A) |
| Sum | 19 | 4 | 19 | 363 (144,219) | 15 | 15 | 7 | 5 | 6 |
| Tehran, Tello: A | X:556324.002  Y:3961538.428 | *Xenopsylla buxtoni* | XBMT1 | 7 (2,5) | OR059306 | OR081769 | PQ203757 (F) | --- | PQ203789 (B) |
| Tehran, Tello: B | X:556533.918  Y:3959718.867 | *Xenopsylla buxtoni* | XBFT2 | 23 (10,13) | OR059307 | OR081770 | --- | PQ203816 (A) | --- |
| Tehran, Tello: C | X:553970.14  Y:3962260.12 | *Xenopsylla buxtoni* | XBMT2 | 24 (12,12) | OR059310 | OR081773 | --- | --- | PQ203794 (B) |
| Tehran, Tello: D | X:556465.018  Y:3959499.669 | *Xenopsylla buxtoni* | XMFT1 | 47 (17,30) | OR059311 | OR081774 | --- | PQ203811 (A) | PQ203793 (B) |
| Tehran, Tello: E | X:556693.409  Y:3959904.746 | *Xenopsylla buxtoni Nosopsyllus iranus iranus* | XMFT3  NIFT1 | 102 (32,68) | OR059340  OR059312 | OR081803  OR081775 | --- | --- | --- |
| Sum | 5 | 2 | 5 | 203 (75,128) | 6 | 6 | 1 | 2 | 3 |
| Bushehr | Borazjan | *Xenopsylla astia* | XAFB1 | 2 (1,1) | OR939694 | PQ164822 | --- | --- | --- |
| Total | 68 | 6 | 68 | 1438 (623,816) | 58 | 58 | 26 | 21 | 26 |

**Table B**. Megablast results for the sequences obtained in this study and the reference species identified from GenBank with the highest identity and minimum genetic distance.

| Species | GenBank (highest identity) | Sample code | Sampling locality | Host | GenBank accession No． | |
| --- | --- | --- | --- | --- | --- | --- |
|  |  |  |  |  | *COII* | *ITS2* |
| *Pulex irritans* | *Pulex irritans* | PI13-14 | Spain | unknown | LR991748 | --- |
|  | *Pulex irritans* | PI33 | Argentina | *Lycalopex griseus* | LR991747 | --- |
| *Pulex irritans* | *Pulex irritans* | SOA1 | Madagascar | unknown | --- | KX982861 |
|  | *Pulex irritans* | 25 | Iran | unknown | --- | OR659511 |
| *Xenopsylla buxtoni* | *Xenopsylla gerbilli minax* | ABL66 | China | unknown | KU880673 | --- |
|  | *Xenopsylla gerbilli minax* | AL01 | China | Rat | MF136074 | --- |
|  | *Xenopsylla conformis conformis* | AL01 | China | Rat | MF136073 |  |
| *Xenopsylla buxtoni* | *Synopsyllus fonquernii* | 244 | Madagascar | unknown | --- | KX982857 |
|  | *Synopsyllus girardi* | 10854 | Madagascar | unknown | --- | KX982858 |
|  | *Xenopsylla cheopis* | Dali | China | unknown | --- | DQ295059 |
|  | *Xenopsylla cheopis* | SNR1 | Madagascar | unknown | --- | KX982860 |
| *Xenopsylla astia* | *Xenopsylla gerbilli minax* | ABL66 | China | unknown | KU880673 | --- |
|  | *Xenopsylla conformis conformis* | AL01 | China | Rat | MF136073 | --- |
| *Xenopsylla astia* | *Xenopsylla cheopis* | Dali | China | unknown | --- | DQ295059 |
|  | *Xenopsylla cheopis* | SNR1 | Madagascar | unknown | --- | KX982860 |
| *Xenopsylla nuttalli* | *Xenopsylla conformis conformis* | AL01 | China | Rat | MF136073 | --- |
|  | *Xenopsylla conformis conformis* | F324 | unknown | unknown | KM890859 | --- |
| *Xenopsylla nuttalli* | *Xenopsylla nuttalli* | Ahar | Iran | unknown | - | OR769686 |
| *Nosopsyllus iranus iranus* | *Nosopsyllus laeviceps* | --- | China | *Rattus norvegicus* | PP838812 | --- |
|  | *Nosopsyllus laeviceps laeviceps* | Alataw11 | China | Rat | MF045767 | --- |
|  | *Nosopsyllus iranus theodori* | F051 | --- | --- | EU335984 | --- |
| *Nosopsyllus iranus iranus* | *Citellophilus sparsilis* | - | unknown | unknown | --- | AY072641 |
|  | *Citellophilus sungaris* | Fl21-01 | Russia | unknown | --- | OL484878 |
|  | *Nosopsyllus barbarus* | NB6.3 | Spain | Rattus sp. | --- | LT703445 |
| *Ctenophthalmus* *rettigi smiti* | *Ctenophthalmus agyrtes* | F401 | unknown | unknown | KM890873 | --- |
|  | *Ctenophthalmus congeneroides congeneroides* | F234 | unknown | unknown | KM890788. | --- |
| *Ctenophthalmus rettigi smiti* | *Ctenophthalmus apertus allani* | CAA1 | Spain | *Arvicola terrestris* | --- | LR594433 |
|  | *Ctenophthalmus baeticus boisseauorum* | CBB9 | Spain | *Arvicola terrestris* | --- | LR594435 |

**Table C**. Genetic distances ± standard deviations (SD) among studied flea species based on 660-732 bp of *COII* gene sequences.

| Species | Mean intergeneric genetic divergence ± SD | | | | | | | | | | |
| --- | --- | --- | --- | --- | --- | --- | --- | --- | --- | --- | --- |
|  | 1 | 2 | 3 | 4 | 5 | 6 | 7 | 8 | 9 | 10 | 11 |
| 1. *Xenopsylla nuttalli* Sarab |  |  |  |  |  |  |  |  |  |  |  |
| 2. *Xenopsylla buxtoni* Akanlu | 0.085±0.011 |  |  |  |  |  |  |  |  |  |  |
| 3. *Xenopsylla buxtoni* Tello | 0.087±0.011 | 0.002±0.001 |  |  |  |  |  |  |  |  |  |
| 4. *Xenopsylla buxtoni* Sarab | 0.085±0.011 | 0.000 | 0.002±0.001 |  |  |  |  |  |  |  |  |
| 5. *Xenopsylla buxtoni* Bukan | 0.085±0.011 | 0.000 | 0.002±0.001 | 0.000 |  |  |  |  |  |  |  |
| 6. *Xenopsylla astia* Busheher | 0.085±0.011 | 0.000 | 0.002±0.001 | 0.000 | 0.000 |  |  |  |  |  |  |
| 7. *Pulex irritans* Akanlu | 0.200±0.018 | 0.194±0.019 | 0.196±0.019 | 0.194±0.019 | 0.194±0.019 | 0.194±0.019 |  |  |  |  |  |
| 8. *Nosopsyllus iranus iranus* Akanlu | 0.239±0.020 | 0.227±0.020 | 0.229±0.020 | 0.227±0.020 | 0.227±0.020 | 0.227±0.020 | 0.208±0.019 |  |  |  |  |
| 9. *Nosopsyllus iranus iranus* Sarab | 0.237±0.020 | 0.229±0.020 | 0.231±0.020 | 0.229±0.020 | 0.229±0.020 | 0.229±0.020 | 0.210±0.019 | 0.002±0.002 |  |  |  |
| 10. *Nosopsyllus iranus iranus* Tello | 0.237±0.020 | 0.225±0.020 | 0.227±0.020 | 0.225±0.020 | 0.225±0.020 | 0.225±0.020 | 0.206±0.019 | 0.002±0.001 | 0.003±0.002 |  |  |
| 11. *Ctenophthalmus rettigi smiti* Sarab | 0.271±0.022 | 0.248±0.022 | 0.250±0.022 | 0.248±0.022 | 0.248±0.022 | 0.248±0.022 | 0.204±0.019 | 0.199±0.019 | 0.201±0.019 | 0.197±0.018 |  |

**Table D**. Genetic distances ± standard deviations (SD) among studied flea species based on 333-486bp of *ITS2* gene sequences.

| Species | Mean intergeneric genetic divergence ± SD | | | | | | | | | | |
| --- | --- | --- | --- | --- | --- | --- | --- | --- | --- | --- | --- |
|  | 1 | 2 | 3 | 4 | 5 | 6 | 7 | 8 | 9 | 10 | 11 |
| 1. *Xenopsylla nuttalli* Sarab |  |  |  |  |  |  |  |  |  |  |  |
| 2. *Xenopsylla buxtoni* Bukan | 0.008±0.006 |  |  |  |  |  |  |  |  |  |  |
| 3. *Xenopsylla buxtoni* Tello | 0.008±0.006 | 0.000 |  |  |  |  |  |  |  |  |  |
| 4. *Xenopsylla buxtoni* Sarab | 0.008±0.006 | 0.000 | 0.000 |  |  |  |  |  |  |  |  |
| 5. *Xenopsylla buxtoni* Akanlu | 0.008±0.006 | 0.000 | 0.000 | 0.000 |  |  |  |  |  |  |  |
| 6. *Xenopsylla astia* Busheher | 0.142±0.025 | 0.137±0.024 | 0.137±0.024 | 0.137±0.024 | 0.137±0.024 |  |  |  |  |  |  |
| 7. *Pulex irritans* Akanlu | 0.178±0.029 | 0.178±0.029 | 0.178±0.029 | 0.178±0.029 | 0.178±0.029 | 0.215±0.033 |  |  |  |  |  |
| 8. *Nosopsyllus iranus iranus* Tello | 0.198±0.030 | 0.198±0.030 | 0.198±0.030 | 0.198±0.030 | 0.198±0.030 | 0.248±0.036 | 0.240±0.036 |  |  |  |  |
| 9. *Nosopsyllus iranus iranus* Sarab | 0.198±0.030 | 0.198±0.030 | 0.198±0.030 | 0.198±0.030 | 0.198±0.030 | 0.248±0.036 | 0.240±0.036 | 0.000 |  |  |  |
| 10. *Nosopsyllus iranus iranus* Akanlu | 0.198±0.030 | 0.198±0.030 | 0.198±0.030 | 0.198±0.030 | 0.198±0.030 | 0.248±0.036 | 0.240±0.036 | 0.000 | 0.000 |  |  |
| 11. *Ctenophthalmus rettigi smiti* Sarab | 0.204±0.032 | 0.209±0.033 | 0.209±0.033 | 0.209±0.033 | 0.209±0.033 | 0.260±0.036 | 0.225±0.034 | 0.171±0.028 | 0.171±0.028 | 0.171±0.028 |  |

**Table E**. Comparison of *Wolbachia* infection prevalence among flea populations by sex, geographic location, and bacterial supergroup.

| Comparison | Groups | Number | Positive (%) | Chi-square | df | p-value |
| --- | --- | --- | --- | --- | --- | --- |
| Sex | Female vs. Male | 53/59 | 73.6%/44.15 | 9.98 | 1 | 0.002* |
| Locations | Tello (Non-endemic) vs Akanlu (Endemic) | 23/69 | 64.28/68.29 | 0.00 | 3 | 0.99 |
|  | Tello (Non-endemic) vs Seyed-Abad (Endemic) | 23/34 | 64.28/38.23 | 0.000 | 1 | 1.000 |
|  | Tello (Non-endemic) vs Sarab (Endemic) | 23/40 | 64.28/37.5 | 0.000 | 1 | 1.000 |
| *Wolbachia* Supergroups | A vs B | 65/65 | 50.76/27.69 | 7.09 | 1 | 0.008* |
|  | A vs F | 65/65 | 50.76/33.84 | 3.82 | 1 | 0.061 |
|  | F vs B | 65/65 | 33.84/50.76 | 0.56 | 1 | 0.45 |


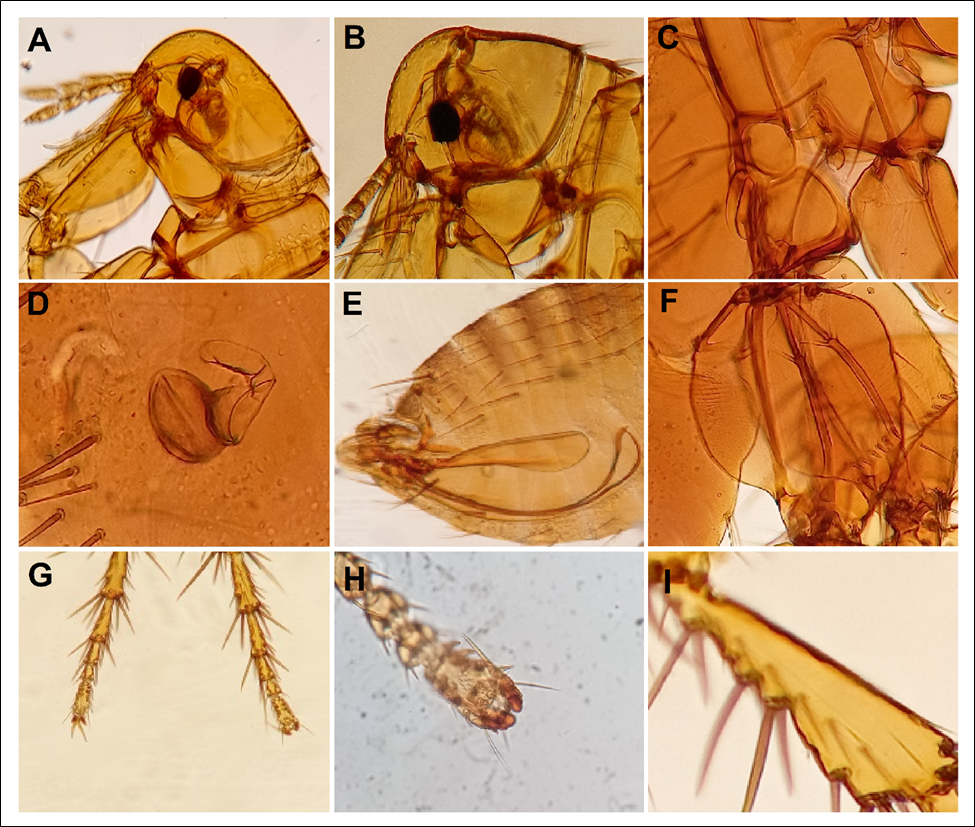


**Fig A.** **Specific identification characters of *Xenopsylla buxtoni*.** A: Female with a smooth anterior margin of the head and without both genal and pronotal combs. B: Male with a shallow occipital groove and a straight ventral outline. C: Mesopleuron of a female with a pleural rod and the suture that separates the sternum from the episternum of the metathorax. D: The oval bulga of spermathecal characterized by a slightly swollen, dark hilla at the base. E: The penis-plate uniformly widened to an obtuse apex, lacking a produced dorso-apical angle. F: Abrupt narrowing of the hind coxa below the middle of the posterior margin. G: The hind tarsus with only one apical bristle on segment II that extends beyond segment IV. H: The male's fore tarsal segment V with two sub-apical plantar spiniform bristles. I: The hind tibia in males with tiny, fine bristles between the fourth and fifth pair of dorsal bristles (Original image by the authors).

**
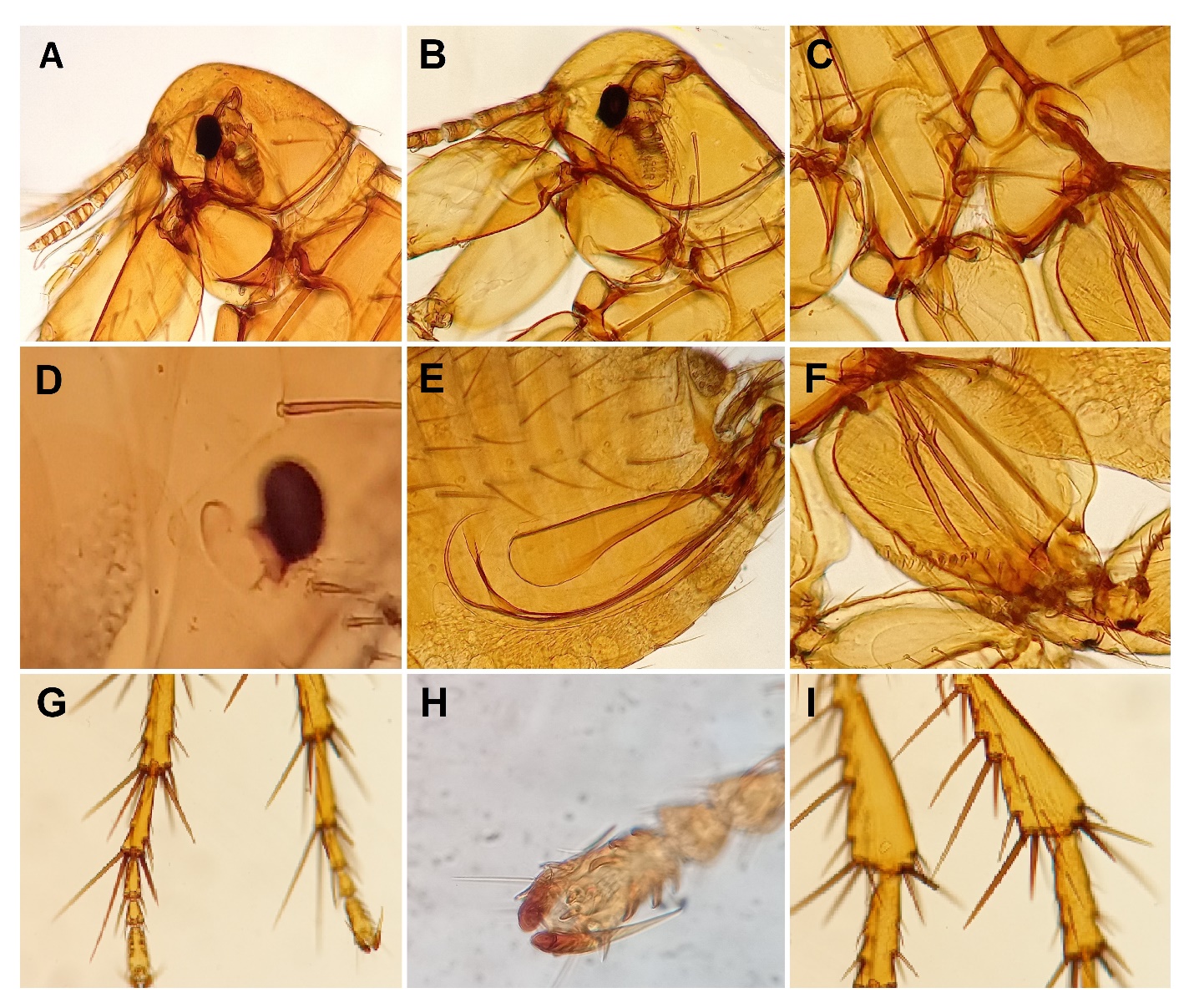
**

**Fig B. Specific identification characters of *Xenopsylla nuttalli***. A: Female with a smooth anterior margin of the head and without both genal and pronotal combs. B: Male with a shallow occipital groove and a straight ventral outline. C: Mesopleuron of a female with a pleural rod and the suture that separates the sternum from the episternum of the metathorax. D: The oval bulga of spermatheca characterized by a slightly swollen, dark hilla at the base. E: The penis-plate uniformly widened to an obtuse apex, without a produced dorso-apical angle. F: Abrupt narrowing of the hind coxa below the middle of the posterior margin. G: The hind tarsus with two apical bristles on segment II extending beyond segment IV. H: The male's fore tarsal segment V with two sub-apical plantar spiniform bristles. (I) The hind tibia in males with tiny, fine bristles between the fourth and fifth pair of dorsal bristles (Original image by the authors).

.


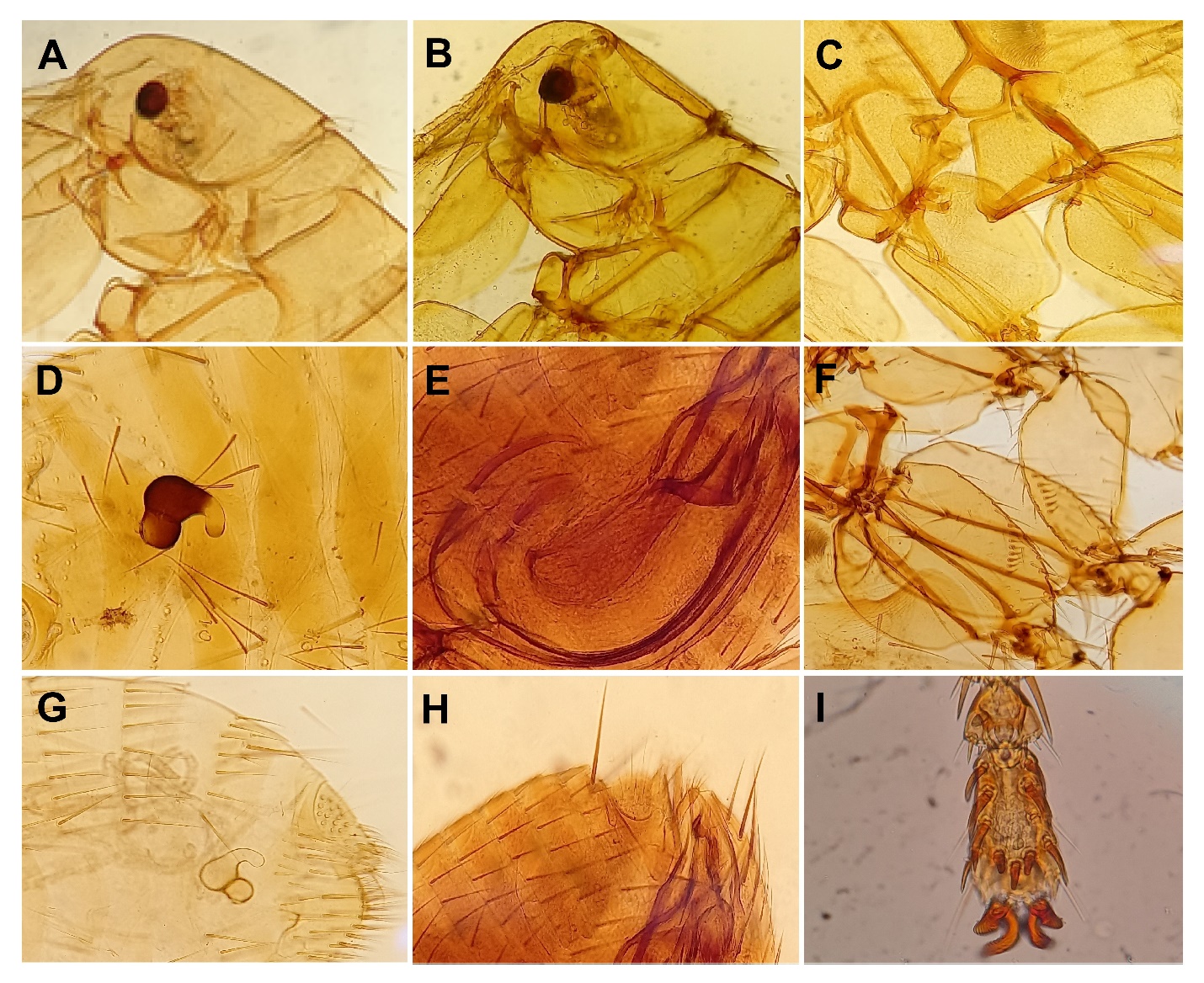


**Fig C. Specific identification characters of *Xenopsylla astia*.** A: Female with a smooth anterior margin of the head and without both genal and pronotal combs. B: Male with a deep occipital groove and undulate ventral outline. C: Mesopleuron of a female with a pleural rod and the suture that separates the sternum from the episternum of the metathorax. D: The subspherical bulga of spermatheca characterized by a swollen hilla at the base twice as wide as the bulga. E: The penis-plate notably broad, with a pronounced convexity at apex, and pre-apically undulating ventral outline. F: Abrupt narrowing of the hind coxa below the middle of the posterior margin. G: Sternum IV-VII of the abdomen often with more than 13 bristles on the two sides together and an outer surface of it. VIII rarely with fewer than 30 including marginal row. H: Sternum VIII typically with 14 to 27 bristles on each side, the antepygidial bristle located submarginally, flanked by smaller bristles. I: The male's fore tarsal segment V with three sub-apical plantar spiniform bristles (Original image by the authors).


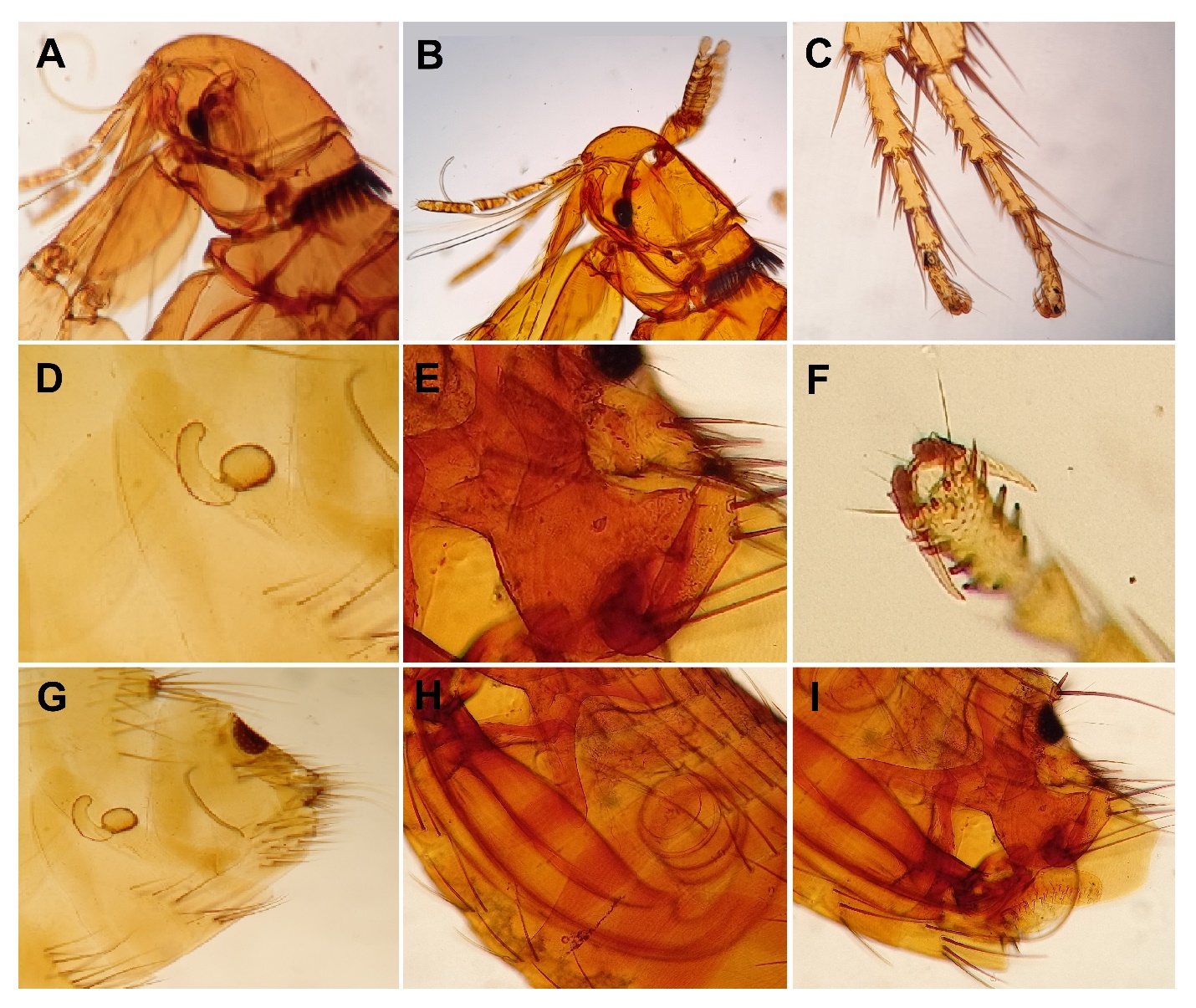


**Fig D. Specific identification characters of *Nosopsyllus iranus iranus***. A: Female with a smooth anterior margin of the head and genal comb absent, while a pronotal comb always present, typically containing less than 24 spines on the two sides together. B: Fracticipit, occipital groove shallow and a straight ventral outline. C: The apical bristle of segment II of the hind tarsus reaching beyond segment IV. D: Spermatheca with a globular bulga, and shorter than the hilla. E: The posterior margin of the fixed process slopes forward in the middle, movable process of clasper slender with triangular apex, acetabular setas arising above the point of articulation of movable process. F: The male's fore tarsal segment V with two sub-apical plantar spiniform bristles. G: The posterior margin of the Sternum VII in the female with two protrusions and a distinct depression. H: The penis plate resembles a dagger, with a sharp tip. I: The apical arm of sternum IX in males with a long beak-shaped projection (Original image by the authors).

.


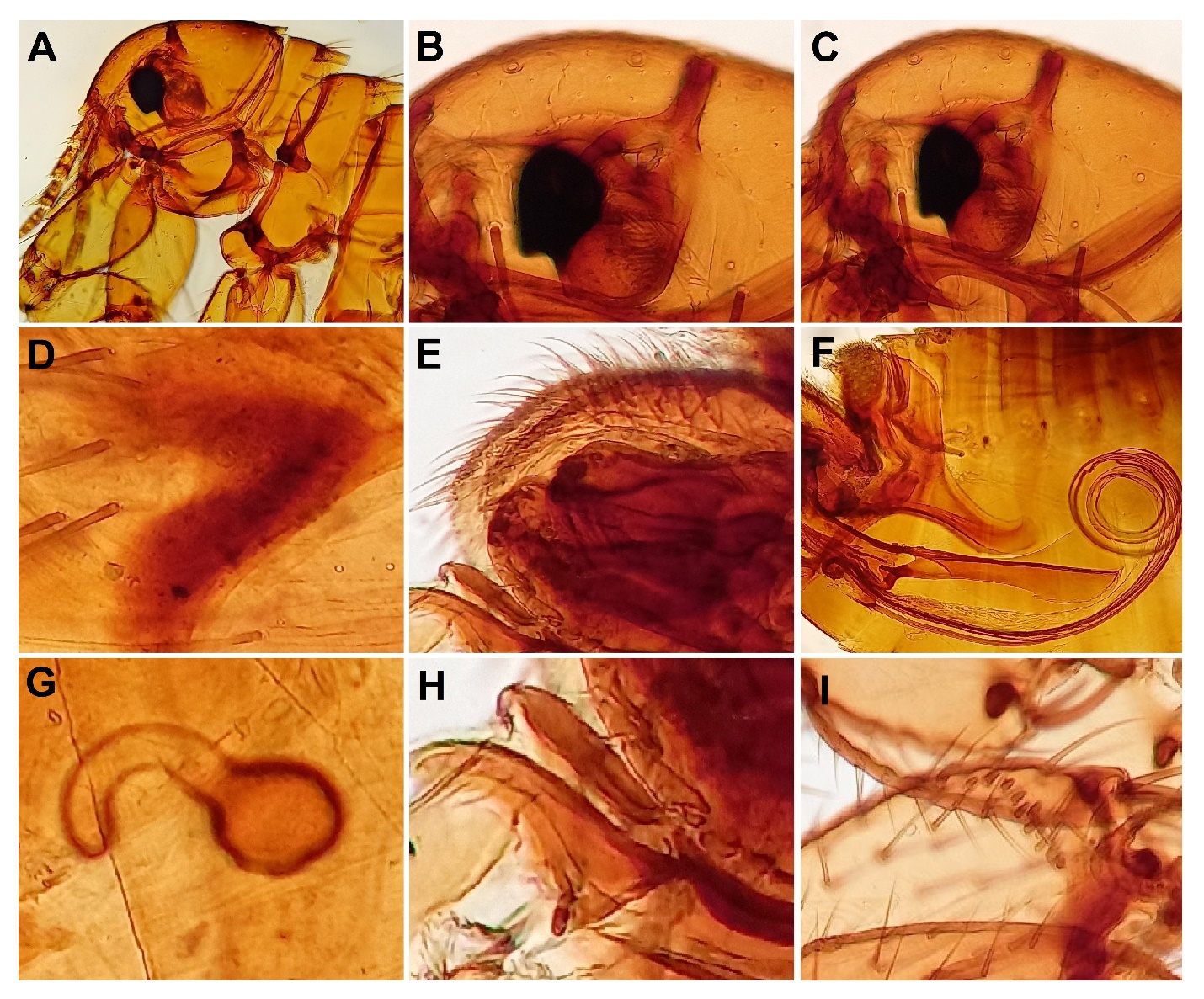


**Fig E. Specific identification characters of *Pulex irritans*.** A: The head with a smooth anterior margin without a tubercle. B: Club of antenna asymmetrical, the anterior segments foliaceous and leaning backwards. C: Genal, pre-ocular, and post-antennal setae of the head. D: Sternite VII of females with a sinus and 4/5 setae on each side. E: Clasper with P1 very large and completely covering P2 and P3, ovoid but with the posterio-distal angle nearly straight; P 2 and P3 about three-quarters length of P1. F: The penis-plate resembles a dagger with a sharp tip. G: The spermatheca with a subglobular bulga and a hilla longer than the bulga. H: Dorsal aedeagal sclerite (das) of males long and slender. I: The ventral margin of the hind coxa with a row of 6-20 spiniform setae near the apex, often irregular and forming a patch (Original image by the authors).

.


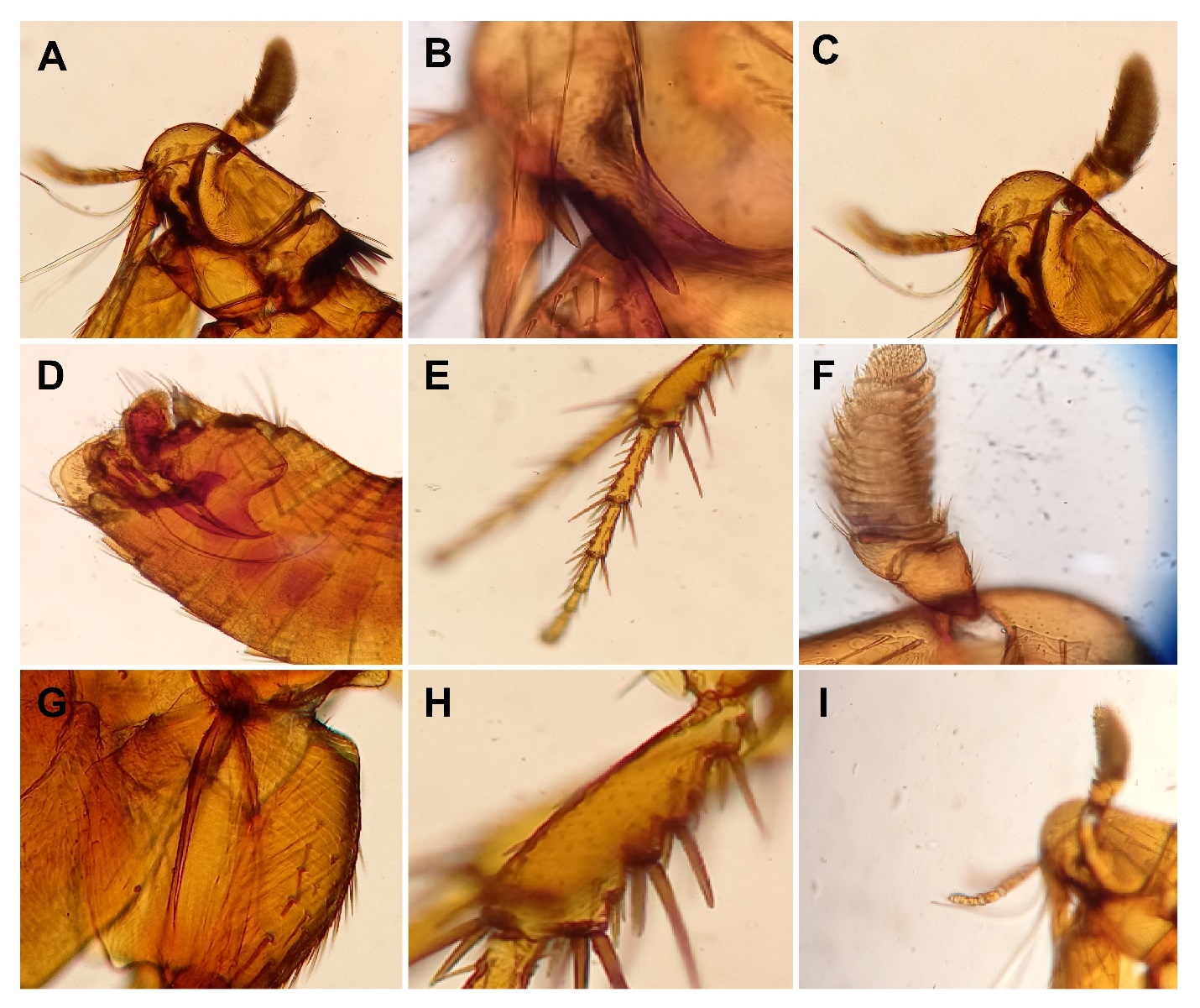


**Fig F. Specific identification characters of *Ctenophthalmus rettigi smiti***. A: The pronotal comb with 18 spines, and the labial palp without a curved apical bristle. B: Genal comb horizontal, with three peg-like spines all of visible in side view and directed obliquely backward. Frons with two rows of bristles. C: The shallow occipital groove and the straight ventral outline in males. The eye is greatly reduced but present in males. D: Fixed process undivided; movable process elongated triangular, with about 10 sensilla along anterior margin, aedeagus with preapical dorsal expansion. E: The apical bristle of segment II of the hind tarsus reaching beyond segment IV. F: The antennal club unmodified and consists of nine segments. G: External coxal ridge present. H: Tooth at apex of hind tibia generally pointed. I: Fracticipit and occiput with three rows of bristles (Original image by the authors).
